# *Enterovirus A* Shows Unique Patterns of Codon Usage Bias in Conventional Versus Unconventional Clade

**DOI:** 10.3389/fcimb.2022.941325

**Published:** 2022-07-14

**Authors:** Liyan Zeng, Ming Chen, Min Wang, Liuyao Zhu, Jingjing Yan, Xiaoyan Zhang, Jianqing Xu, Shuye Zhang

**Affiliations:** ^1^ Shanghai Public Health Clinical center AND Institutes of Biomedical Sciences, Fudan University, Shanghai, China; ^2^ Clinical Center for BioTherapy & Institutes of Biomedical Sciences, Zhongshan Hospital, Fudan University, Shanghai, China

**Keywords:** *enterovirus A*, HFMD, codon usage, natural selection, evolution

## Abstract

*Enterovirus A* (*EV-A*) species cause hand, foot and mouth disease (HFMD), threatening the health of young children. Understanding the mutual codon usage pattern of the virus and its host(s) has fundamental and applied values. Here, through examining multiple codon usage parameters, we found that the codon usage bias among *EV-A* strains varies and is clade-specific. EVA76, EVA89, EVA90, EVA91 and EVA92, the unconventional clade of *EV-A* strains, show unique codon usage pattern relative to the two conventional clades, including EVA71, CVA16, CVA6 and CVA10, *etc.* Analyses of Effective Number of Codon (ENC), Correspondence Analysis (COA) and Parity Rule 2 (PR2), *etc.*, revealed that the codon usage patterns of *EV-A* strains are shaped by mutation pressure and natural selection. Based on the neutrality analysis, we determined the dominant role of natural selection in the formation of the codon usage bias of *EV-A*. In addition, we have determined the codon usage compatibility of potential hosts for *EV-A* strains using codon adaptation index (CAI), relative codon deoptimization index (RCDI) and similarity index (SiD) analyses, and found that *EV-A* showed host-specific codon adaptation patterns in different clades. Finally, we confirmed that the unique codon usage pattern of the unconventional clade affected protein expression level in human cell lines. In conclusion, we identified novel characteristics of codon usage bias in distinct *EV-A* clades associated with their host range, transmission and pathogenicity.

## 1 Introduction

Enterovirus (EV) is a small, non-enveloped RNA virus of the family *Picornaviridae*, consisting of a positive-sense single-stranded RNA genome of approximately 7.4 kb and a 30 nM icosahedral capsid. The EV genome has an open reading frame, and the encoded polyprotein consists of P1, P2 and P3 regions. The P1 region encodes the capsid proteins: VP1, VP2, VP3 and VP4, while the P2 and P3 regions (P2-P3) encode the non-structural proteins: 2A, 2B, 2C, 3A, 3B, 3C and 3D. Four species of EVs are known to infect humans: *EV-A, EV-B, EV-C* and *EV-D*. *EV-A* consists of at least 22 serotypes (Oberste and Gerber, 2014). EVA71, CVA6, CVA10 and CVA16 in *EV-A* are the major pathogens of HFMD, although other serotypes of *EV-A* and some *EV-B* strains are also less frequently identified (Zhuang et al., 2015). HFMD mainly affects children less than 5 years of age worldwide. Typical symptoms of HFMD include fever, nodular lesions and small ulcers or blisters in the hands, feet and mouth, *etc* (Organization, W.H., 2011). Most affected children can recover quickly while a small number of children can have serious complications such as myocarditis, pulmonary edema, aseptic meningoencephalitis and even death. It has been reported that CVA16 infection is benign and self-limited (Mao et al., 2014) while EVA71 infection can cause serious illnesses (Wang and Liu, 2014). Recently, CVA6 and CVA10 have increasingly caused outbreaks of HFMD around the world as emerging pathogens of HFMD (Bian et al., 2015; Zhang et al., 2017). The CVA6-related HFMD showed a higher incidence among adults (Bian et al., 2015). CVA10 is often co-epidemic with CVA6 (Blomqvist et al., 2010) and the co-infection of multiple pathogens increases the risk of virus recombination (Liu et al., 2014). EVA76, EVA89, EVA90 and EVA91 have been characterized as an unique phylogenetic clade of *EV-A* since 2005 (Oberste et al., 2005; Xu et al., 2011; Tao et al., 2013). Based on the phylogenetic and receptor usage, *EV-A* strains are currently divided into three unique clades. Clade 1 includes EVA71 and CVA16 using SCARB2 as receptor (Yamayoshi et al., 2009), clade 2 includes CVA6 and CVA10 using KREMEN1 as receptor (Staring et al., 2018), whereas clade 3 includes EVA76 and EVA89 with their receptor still unidentified. Recently, we further showed that the *EV-A* strains of EVA76 and EVA89 clade have virological properties distinct from other “conventional” *EV-A* strains (Wang et al., 2020), and are regarded as “unconventional” *EV-A* viruses. It is concerned that the application of EVA71 vaccine may alter the spectrum of the HFMD epidemic, and that co-circulating of *EV-A* viruses may results in new mutants or recombinants (Yip et al., 2013; Lukashev et al., 2014). Unconventional *EV-As* has the potential to spread and cause outbreaks in the future. Therefore, further investigation into the evolution and variability of the *EV-A* gene pools is required.

This codon usage bias has been observed in all branches of life and results in species-specific patterns of codon usage (Sharp et al., 1995). The cause of codon bias may be explained by mutation pressure and natural selection pressure (Bulmer, 1991). Mutation pressure theory explains codon bias through genomic mutation bias, where the composition of AU/GC nucleotides is uneven (Sueoka, 1988). Natural selection theory assumes that the codon usage bias comes mainly from translational pressure, due to the host tRNA pool and secondary RNA structure (Simmonds et al., 2004; Steil and Barton, 2009). The CpG and UpA composition of pathogens may also be classified as selective pressure as a result of immune defense recognition (Belalov and Lukashev, 2013). The codon usage bias are important for the fitness, evolution and virulence of viruses. Discovering codon usage patterns of viruses may provide insights into the drivers of virus evolution and adaptation.

Although there are previous reports regarding the codon usage patterns for some major pathogens of HFMD (Ma et al., 2014; Zhang et al., 2014), the patterns of the *EV-A* species are still unclear, especially for the unconventional strains (Liu et al., 2011; Su et al., 2017). The difference of codon bias between the structural and non-structural genetic regions of *EV-A* is also uncertain. Consequently, in this study, we conducted a wide range of analyses to investigate (i) the codon bias pattern of *EV-A*; (ii) the primary factors causing codon usage bias of *EV-A*; (iii) the adaptability of *EV-A* to the host; and (iv) the level of protein expression affected by the specific codon bias.

## 2 Materials and Methods

### 2.1 Viral Sequence Data

The complete genomic sequences and coding sequence (CDS) of 1006 *EV-A* strains were obtained from the Virus Pathogen Resource (ViPR) database (Pickett et al., 2012).

### 2.2 Recombination and Phylogenetic Analyses

Phylogenetic analysis requires that the evolution of a serious of sequences may be properly delineated by a single phylogenetic tree, whereas recombination events in these sequences may severely compromise the accuracy of phylogenetic trees. Hence, non-recombinant datasets produced by recombination analysis are desirable prior to phylogenetic analysis. We have conducted the recombination analysis by Recombination Detection Program (version 4.95) using RDP methods (Martin et al., 2015). Phylogenetic analysis was then carried out on non-recombinant sequences in the MEGA software (version 7.0) using the maximum likelihood model (bootstrap value=1000) (Kumar et al., 2016).

### 2.3 Nucleotide Composition Analysis

The codon compositions in 3rd position (A3%, U3%, C3% and G3%) have been calculated with Codon W 1.4.2 (Peden and John, 2000). The frequencies of A, U, C and G (%), GC/AU and GC1, GC2, GC12, GC3 content (frequency of occurrence of GC-dinucleotides at each location) have also been calculated. Five codons were excluded from the codon usage bias analyses, including stop codons (UAA, UAG and UGA) and the only codon encoding for Met(AUG) and Trp(UGG).

### 2.4 Dinucleotide Analysis

The frequencies of dinucleotides are known to be influenced by corresponding individual nucleotide abundance. To avoid this bias, the odds ratio index (ρXY) is used (Karlin et al., 1997). X and Y stand for each nucleotide (A, U, G or C). F_X_ is the frequency of X while F_Y_ is the frequency of Y. F_XY_ is the frequency of dinucleotide XY. The calculation of ρXY is as follows:


$$\text{ρXY}={\text{F}}_{\text{XY}}/{\text{F}}_{\text{X}}{\text{F}}_{\text{Y}}$$

ρXY=1 indicates that the dinucleotide is present at the corresponding single nucleotide frequencies. Values of ρXY>1 indicate an over-representation of dinucleotide, and ρXY<1 indicates an under-representation.

### 2.5 Relative Synonymous Codon Usage Analysis

The relative synonymous codon usage (RSCU) values for all the *EV-A* CDS were calculated to determine the characteristics of synonymous codon usage. The RSCU values represent the ratio of the observed frequency of one codon to its expected frequency in the synonymous codon family, considering that all codons for an particular amino acid are equally used. Thus, the RSCU values may eliminate the influences from the amino acid compositions or the coding sequence sizes. When the RSCU value is 1.0, there is no bias for codon usage (Sharp and Li, 1986). Synonymous codons with RSCU values >1.0 display a positive codon usage bias while those with RSCU values <1.0 display a negative codon usage bias. Synonymous codons with RSCU values above 1.6 and below 0.6 were considered to be over-represented or under-represented codons, respectively (Wong et al., 2010). The RSCU of individual codons has been calculated by CodonW 1.4.2.

### 2.6 Effective Number of Codons Analysis

The effective number of codons (ENC) value describes the degree of unbalanced use of synonymous codons (Wright, 1990). ENC values range between 20 and 61. The lower ENC value indicates a greater extent of codon bias within a gene. Typically, an ENC value below 35 denotes a strong codon bias while an ENC value above 50 denotes a weak codon bias. ENC values were calculated through CodonW 1.4.2.

The ENC-GC3 may be used to explore the pattern for synonymous codon usage. Genes where the codon selection is limited only by the G&C mutation will be on or just below the the expected ENC curve (Comeron and Aguadé, 1998).

### 2.7 Correspondence Analysis

Correspondence analysis (COA) is a multivariate analysis that provides a geometrical representation of a contingency table, which is used to analyze the major trends of codon usage patterns among genes (Grantham et al., 1980). RSCU is used in COA to minimize the effect of amino acid composition on the analysis of codon usage. Each coding sequence is represented as a vector with 59 dimensions which correspond to the RSCU value of each codon except for Met, Trp and the stop codons. COA reduces the high-dimensional codon-frequency data to a limited number of variables, referred to as principal axes. The axes preserve much of the information about the variability of codon usage among the genes. COA plot selects the first two principal axes as X and Y axes and shows the viral strains as scatters in the two-dimensional diagram. COA analysis is conducted using CodonW 1.4.2.

### 2.8 Correlation Analysis

Correlation analyses were conducted to identify the relationships between the nucleotide composition and the first two COA principal axes using Spearman’s rank correlation test, using the R package “Corrgrams” (Friendly, 2002).

### 2.9 Parity Rule 2 Analysis

Parity rule 2 (PR2) analysis, exploring the bias between A3/(A3 + U3) and G3/(G3 + C3) in the amino acid family with four-codon, was used to demonstrate the effects of mutation pressure and natural selection on the codon usage. The dots at the center of the plot (A=U, G=C) indicate equal effects of mutation and selection (Sueoka, 1995; Sueoka, 1999).

### 2.10 Neutrality Plot Analysis

In the neutrality plot analysis, the regression coefficient (CG12 against GC3) is considered to be the mutation-selection equilibrium coefficient. This analysis was performed to determine the effects of mutation pressure and natural selection on the codon usage patterns of the *EV-A* coding sequences (Sueoka, 1988). The slope of the regression lines (ϵ) may indicate the evolutionary rates of mutation pressure, whereas 1-ϵ represents selective pressure. If ϵ is close to 1, it means a strong correlation between GC12 and GC3, indicating that mutation pressure is dominant. While ϵ is near 0, it indicates a low mutation pressure.

### 2.11 Codon Adaptation Analysis

The codon adaptation index (CAI) is a common measure used to quantify the similarity of the synonymous codon usage between samples and reference. It may also be used to predict the gene expression level based on its codon sequence. The CAI values range from 0 through 1. The higher the CAI value, the higher the degree of gene expression and the higher the adaptation to specific hosts (Sharp and Li, 1987). We also borrowed two other indices reported in recent studies, the relative codon deoptimization index (RCDI) and the similarity index (SiD), to confirm the effects of *EV-A* codon usage patterns on hosts or vectors. When the RCDI value is closer to 1, the codon usage pattern between the virus and its hosts is more similar. When RCDI is greater than 1, the higher RCDI value indicates greater deoptimization of the virus codon usage patterns relative to hosts (Mueller et al., 2006). The SiD value ranges from 0 to 1. When the SiD value is closer to 1, the codon usage patterns between the virus and the hosts differ significantly, and the effect of host codon usage on the virus is stronger (Zhou et al., 2013).

The CAI and RCDI analysis of the *EV-A* coding sequences was carried out using the CAIcal server (Puigbò et al., 2008). Here, synonymous codon usage patterns of selected potential hosts were used as references and obtained from the Codon Usage database (Nakamura et al., 2000).

### 2.12 Codon Optimization Experiment

*EV-A* genomic sequences were obtained from the National Center for Biotechnology Information, including EV71 (KU936132), CVA6 (KX064292), CVA10 (AY421767), CVA16 (MG957117), EVA76 (AY697458.1) and EVA89 (KT277550.1). The viral capsid encoding segment was obtained through *in vitro* synthesis, and was cloned into the expression vector by a Seamless Cloning Kit (D7010M, Beyotime, Shanghai, China) according to the manufacturer’s instruction. The expression vector was derived from the previously reported “pcDNA6.0A-FY-capsid-GFP” (Chen et al., 2012). The vector is based on pcDNA6.0 backbone and contained a CMV promoter. A fused green fluorescent protein (EGFP) was introduced between the promoter and the viral capsid, and a EVA71 2A protease recognition site (-AITTL-) was inserted between EGFP and VP4. Thus, EGFP was fused to the capsid protein and co-expressed. The amount of green fluorescence could be employed to signal the production of capsid protein.

## 3 Results

### 3.1 Recombination and Phylogenetic Analyses of EV-A Genomes

To avoid the interference by the recombination events in phylogenetic analysis, recombination analysis was first performed on 1006 *EV-A* genomes to obtain non-recombinant datasets. 878 sequences showing a recombinant signal and 3 containing several stop codons were excluded from the analysis. The P1 region of 125 *EV-A* genomes and three EVs as outgroup (EVD68, accession numbers: AY426531, CVB3, accession numbers: M33854, PV1, accession numbers: V01150) were subjected to a phylogenetic analysis. A maximum likehood (ML) tree was built, and verified with 1000 bootstrap replicates. *EV-A* clustered into three phylogenetic clades: clade 1 (n=36), clade 2 (n=80) and clade 3 (n=9). The clade 1 includes CVA7, CVA14, CVA16, EVA71 and EVA120. Clade 2 includes CVA2, CVA3, CVA4, CVA5, CVA6, CVA8, CVA10 and CVA12. Clade 3 comprises EVA76, EVA89, EVA90, EVA91 and EVA92. The descriptions of the 125 strains are given in **Supplementary Table S1** and the phylogenetic tree is shown in **Supplementary Figure S1**.

### 3.2 The Genomes of EV-A Are Rich in AU Nucleotides and Lack CpG Dinucleotide

To understand the common features and differences of the *EV-A* genomes, we determined the contents of nucleotides and dinucleotides (**Supplementary Table S2**). The composition for nucleotide A (28.44 ± 0.35%) is highest, followed by U (24.49 ± 0.32%), C (24.23 ± 0.32%), and G (22.84 ± 0.38%). The proportions of different nucleotides at the 3rd codon position (A3, U3, G3 and C3) show that U3 (29.04 ± 1.04%) and C3 (27.04 ± 0.86%) are higher than A3 (24.65 ± 0.72%) and G3 (19.26 ± 0.90%). Compositions for GC and AU are 47.07 ± 0.61% and 52.93 ± 0.61% respectively, while compositions for GC3 and AU3 are 46.31 ± 1.60% and 53.69 ± 1.59%. These data show that *EV-A* coding sequences are rich in AU. The Kruskal-Wallis test, one way ANOVA and multiple comparison by LSD test was performed on three EV-A clades for each nucleotide index (**Supplementary Table S3**). Three of the 15 nucleotide indices show significant differences across the three *EV-A* clades (p<0.01). Other comparisons show significant differences between clade 1 and clade 2 in 7 out of 15 indices (p<0.05), whereas clade 3 shows significant differences in 15 indices compared to clade 1 and clade 2 (p<0.01).

**Figure 1:**
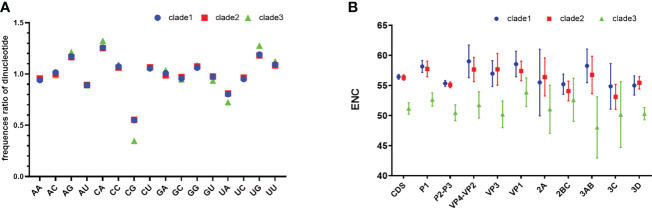
Relative dinucleotide analysis and ENC plot. **(A)** Dinucleotide composition of EV-A clades. **(B)** Clade-level comparison of ENC values of *EV-A* CDS and individual gene segments. CDS is short for coding sequences.

Analysis of dinucleotide shows that the relative frequency of CpG is significantly lower than other dinucleotides (**Figure 1A**), which explains in part why G and G3 are less frequent. The composition of UpA is also significantly lower than that of other dinucleotides. CpG levels for clade 3 are significantly lower than other clades (p <0.05).

### 3.3 Codon Usage Bias Varies Among Different EV-A Clades

We found that clade 3 has a particular nucleotide composition that may affect codon bias. To estimate the extent of the codon usage bias in *EV-A* coding sequences, the ENC values were calculated (**Figure 1B**). The average ENC value of all *EV-A* strains is 55.97 ± 1.43, while those of clade 1, 2 and 3 are 56.44 ± 0.41, 56.30 ± 0.45, 51.16 ± 0.96. For the CDS of each gene, the ENC values of clade 3 were significantly lower than the other two clades (p<0.01), while the ENC values of clade 1 and clade 2 are similar. We also noticed that ENC values of P2P3 regions were significantly lower than P1 (p<0.01). Overall, ENC values indicated that the codon usage bias in *the EV-A* genomes varied in different clades.

### 3.4 EV-A Genomes Have Evolved Clade-Specific RSCU Patterns

RSCU was calculated to determine the synonymous codons usage in the *EV-A* coding sequences (**Table 1**) and the preferred codon with the highest RSCU value for each animo acid was bolded. We noted that the commonly/uncommonly preferred codons ratios among *EV-A* clade 1:clade 2, clade 1:clade 3 and clade 2:clade 3 were 14:4, 12:6 and 12:6, respectively. By comparing the codon usage patterns of *Homo sapiens* and *EV-A*, the ratio of coincident/antagonistic preferred codons is 7/11 in clade 1, 9/9 in clade 2, while only 4/14 in clade 3 among the 18 preferred codons (**Table 1**). This clade-specific RSCU pattern highlights the distinct evolutionary dynamics of the *EV-A* clades.

**Table 1:** The relative synonymous codon usage (RSCU) patterns of Enterovirus A.

Amino acid	Codon	*Enterovirus A*				Host
		Overall	Clade 1	Clade 2	Clade 3	H. sapiens
Phe	**UUU**	**1.06**	**1.06**	**1.06**	**1.02**	0.93
	UUC	0.94	0.94	0.94	0.98	**1.07**
Leu	UUA	0.77	0.96	0.66	0.98	0.46
	**UUG**	**1.30**	**1.28**	**1.34**	1.05	0.77
	CUU	1.08	0.97	1.12	**1.19**	0.79
	CUC	1.09	1.02	1.11	1.13	1.17
	CUA	0.87	0.83	0.89	0.89	0.43
	CUG	0.89	0.95	0.88	0.76	**2.37**
Ile	**AUU**	**1.15**	**1.19**	1.09	**1.41**	1.08
	AUC	1.14	1.03	**1.20**	1.09	**1.41**
	AUA	0.71	0.78	0.70	0.57	0.51
Val	GUU	0.91	0.86	0.90	1.20	0.73
	GUC	0.82	0.85	0.78	0.99	0.95
	GUA	0.69	0.67	0.70	0.59	0.47
	**GUG**	**1.58**	**1.61**	**1.61**	**1.22**	**1.85**
Pro	CCU	1.19	1.02	1.30	0.93	1.15
	CCC	0.90	1.01	0.86	0.82	**1.29**
	**CCA**	**1.52**	**1.46**	**1.49**	**2.00**	1.11
	CCG	0.39	0.51	0.35	0.25	0.45
Thr	**ACU**	**1.23**	**1.23**	1.21	**1.39**	0.99
	ACC	1.20	1.21	1.22	1.07	1.42
	ACA	1.23	1.19	**1.23**	1.38	**1.14**
	ACG	0.33	0.37	0.33	0.17	0.46
Ala	**GCU**	**1.36**	**1.36**	**1.35**	**1.49**	1.06
	GCC	0.95	1.07	0.91	0.87	**1.60**
	GCA	1.30	1.17	1.35	1.47	0.91
	GCG	0.39	0.40	0.40	0.19	0.42
Tyr	UAU	0.96	**1.03**	0.93	0.93	0.89
	**UAC**	**1.04**	0.97	**1.07**	**1.07**	**1.11**
Ser	UCU	1.09	1.06	1.10	1.18	1.13
	UCC	1.05	1.11	1.02	1.06	1.31
	**UCA**	**1.29**	**1.29**	**1.24**	**1.74**	0.90
	UCG	0.42	0.45	0.43	0.24	0.33
	AGU	1.19	1.17	1.21	1.16	0.90
	AGC	0.95	0.92	1.00	0.63	**1.44**
Arg	**AGA**	**2.00**	**2.16**	**1.80**	**3.24**	**1.29**
	CGU	0.44	0.45	0.45	0.34	0.48
	CGC	1.01	0.90	1.12	0.39	1.10
	CGA	0.57	0.52	0.63	0.21	0.65
	CGG	0.32	0.36	0.31	0.15	1.21
	AGG	1.66	1.61	1.68	1.68	1.27
Cys	UGU	0.99	0.90	**1.01**	**1.09**	0.91
	**UGC**	**1.01**	**1.10**	0.99	0.91	**1.09**
His	CAU	0.79	0.75	0.78	0.93	0.84
	**CAC**	**1.21**	**1.25**	**1.22**	**1.07**	**1.16**
Gln	**CAA**	**1.15**	**1.09**	**1.16**	**1.22**	0.53
	CAG	0.85	0.91	0.84	0.78	**1.47**
Asn	AAU	0.97	0.95	0.97	**1.03**	0.94
	**AAC**	**1.03**	**1.05**	**1.03**	0.97	**1.06**
Lys	AAA	0.88	0.94	0.82	**1.14**	0.87
	**AAG**	**1.12**	**1.06**	**1.18**	0.86	**1.13**
Asp	**GAU**	**1.13**	**1.06**	**1.16**	**1.19**	0.93
	GAC	0.87	0.94	0.84	0.81	**1.07**
Glu	GAA	0.95	0.88	0.97	**1.07**	0.84
	**GAG**	**1.05**	**1.12**	**1.03**	0.93	**1.16**
Gly	**GGU**	**1.12**	**1.14**	**1.09**	**1.32**	0.65
	GGC	0.83	0.85	0.81	0.82	**1.35**
	GGA	1.02	0.94	1.04	1.20	1.00
	GGG	1.03	1.06	1.05	0.65	1.00

Preferred codons of EV-A and H. sapiens are shown in bold.

**Figure 2:**
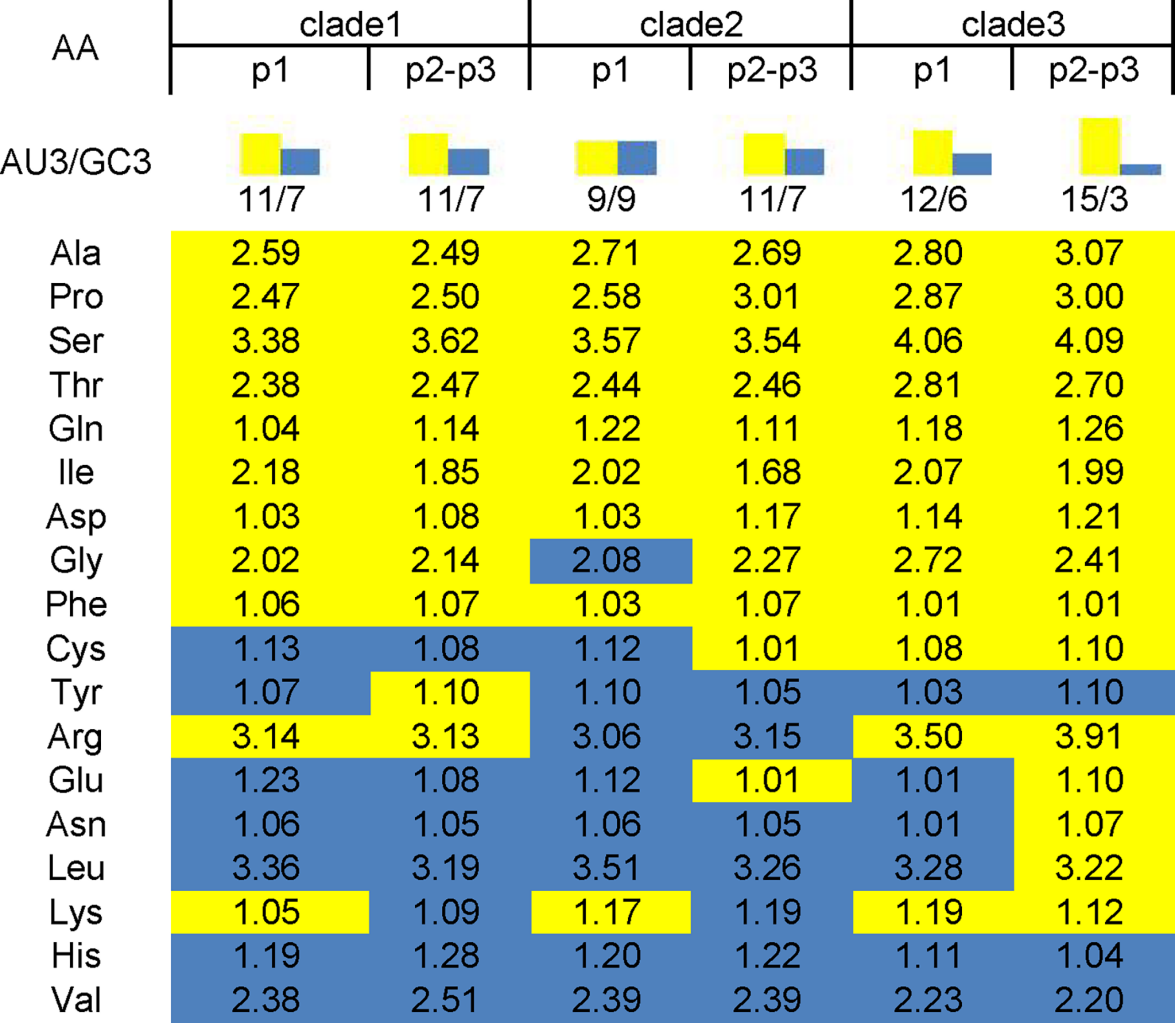
AU3 and GC3 in codons of P1 and P2P3 regions of *EV-A* clade 1, clade 2 and clade 3. Numbers in the square array indicate the RSCU values of preferred codon by each amino acid. Yellow shade indicate AU3 and blue shade indicate GC3 for the codon ending.

Of the 18 preferred codons, eight are G/C-ended (four C-ended; four G-ended) and the remaining ten are A/U-ended (five A-ended; five U-ended) in the *EV-A* coding sequences. The findings show that *EV-As* prefer A/U at the end of codons rather than G/C. We have summarized the RSCU values of the preferred codons by each amino acid in **Figure 2**. It also shows that *EV-As* prefer A/U at the end of codons rather than G/C. We noted that *EV-A* clade 3 has a stronger AU3 bias than other clades, and the AU3 bias in the P2P3 region of *EV-As* is stronger than the P1 region.

### 3.5 Trends in Codon Usage Variation of EV-A

**Figure 3:**
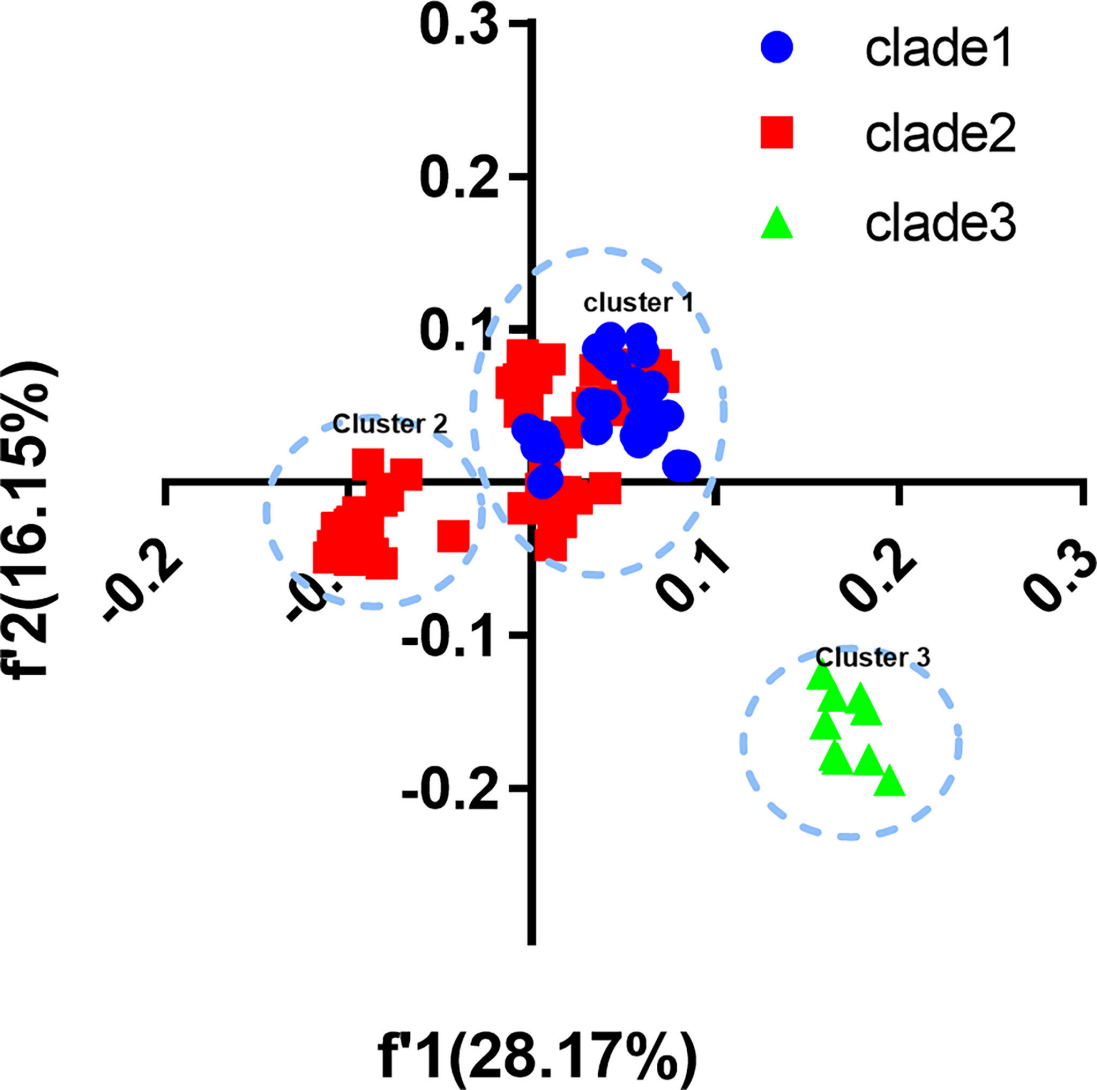
Correspondence analysis (COA). COA plots were constructed for coding sequences of each *EV-A* strains.

We built COA plots of CDS and each gene segment to show trends in codon usage variations among different EV-A strains (**Figure 3**). The first (f’1) and second (f’2) principal axes represent respectively 28.17% and 16.15% of the total variation of the *EV-A* coding sequences. The *EV-A* strains were grouped into three distinct clusters on the plot which were shown to be largely correlated with their phylogenetic relationships. Overall, clade 1 and clade 2 formed cluster I, except for CVA5 and CVA6, which formed cluster II; and cluster III comprised all clade 3 strains. These data further support that clade 3 has a unique codon usage pattern. At the level of the individual genes, the clade-specific clustering was also observed (**Supplementary Figure S2**). For genes encoding 3C, 3D and VP3, clade 3 strains were clustered independently, indicating the effect of divergence events on individual genes. For the genes encoding 2A, 2BC, 3AB, VP1, VP4 and VP2, overlapping patterns in codon usage were observed. Based on these data and together with ENC and RSCU, we confirm that clade 3 has a unique codon usage pattern and likely has an independent evolutionary origin. Conversely, the overlap between clade 1 and clade 2 indicates that they have similar codon usage patterns and a closer relationship. Together, clade 3 stands apart from other clades, which may be affected by unique evolutionary forces.

### 3.6 Codon Usage Patterns of EA-V Strains Are Influenced by Mutation Pressure and Natural Selection

**Figure 4:**
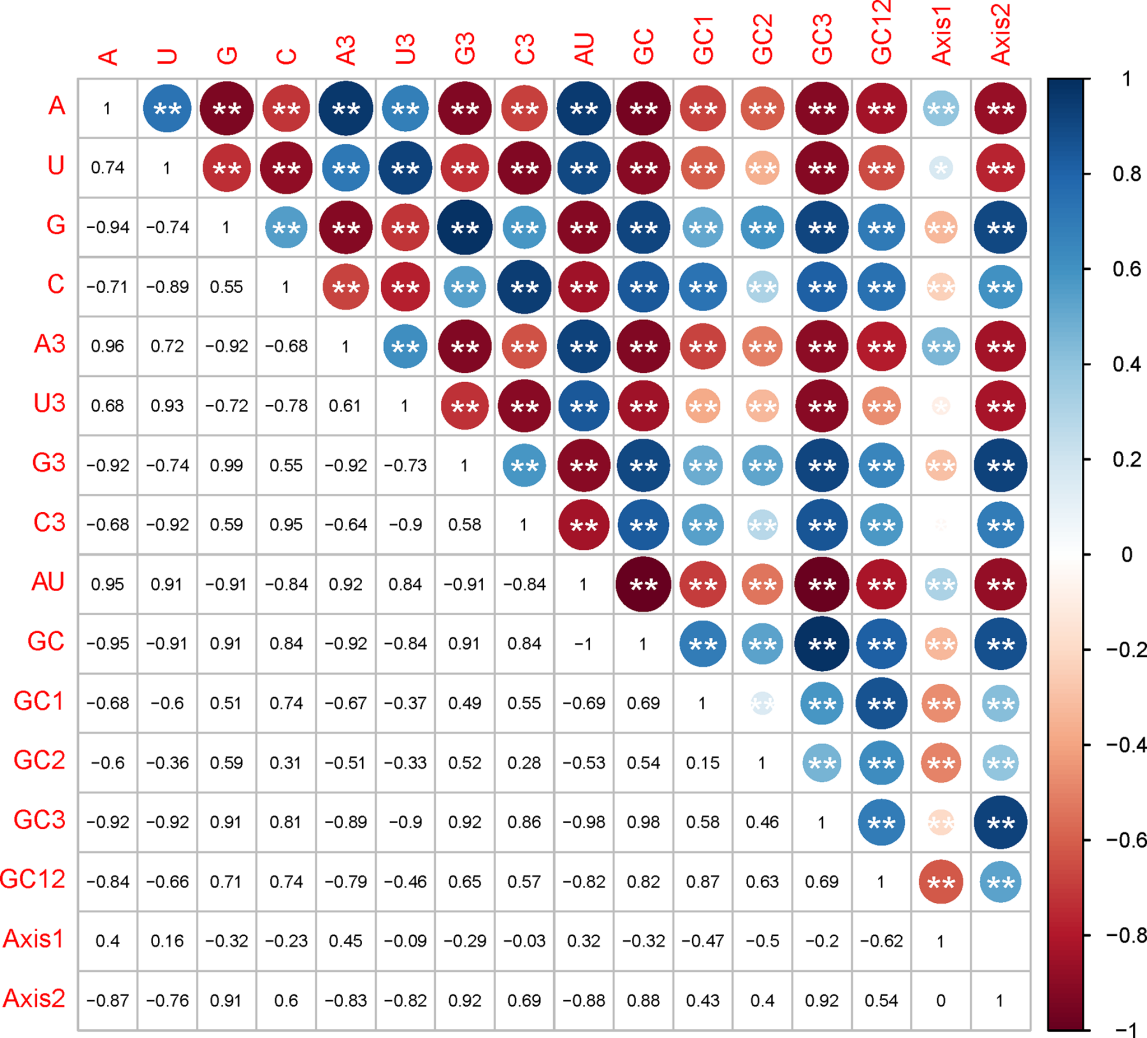
Correlation analysis between nucleotide compositions and the two principal axes of COA plot. The numbers in lower triangle matrix show the slope of regression line. The upper triangle matrix shows the information by the color and size of circles; Dark blue means positive correlation and dark red means negative correlation; The bigger circle means more significant correlation; NS means non-significant (p > 0.05), * represents p < 0.05, ** represents p < 0.01.

Mutational pressure and translational selection were reported as the primary factors influencing the codon usage bias. Here, we examined whether they have affected the *EV-A* codon usage and which factor is dominant through correlation analysis, PR2 plot and ENC-GC3 plot analysis. In the correlation analysis, we compared the correlation between the nucleotide compositions and the principal axes of the COA plot, and noted a significant positive or negative correlation between nucleotide composition, such as A/A3 or A/G. We also observed that most nucleotide composition constraints were significantly correlated with f’1 and f’2 (**Figure 4**). Together, these results supports that mutation pressure from the nucleotide composition may have affected the *EV-A* codon biases.

**Figure 5:**
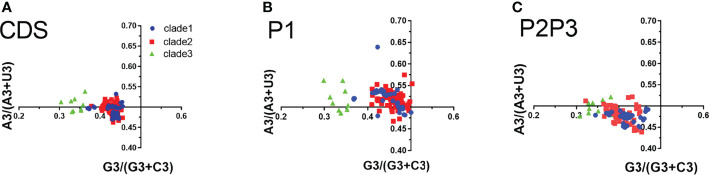
Parity rule 2 (PR2) bias plots. PR2 plots were constructed for the coding sequences, P1 and P2P3 regions of *EV-A* strains respectively. **(A)** coding sequences, **(B)** P1 region, **(C)** P2P3 region. The blue dots, red squares, and green triangles indicate clade 1, clade 2 and clade 3 strains.

In PR2 plot, the relationship between A/U and G/C composition on the 3rd codon position in four degenerated codon families (Ala, Arg, Gly, Leu, Pro, Ser, Thr, and Val) was analyzed. We observed that U3 was used more frequently than A3, while G3 is approximately equal to C3 in the entire coding sequences (**Figure 5**). G3 and U3 were more frequent in P1 regions, while C3 and U3 were more frequent in P2-P3 regions. The A3/U3 and G3/C3 bias denotes the influence of both selective pressure and mutation effect.

**Figure 6:**
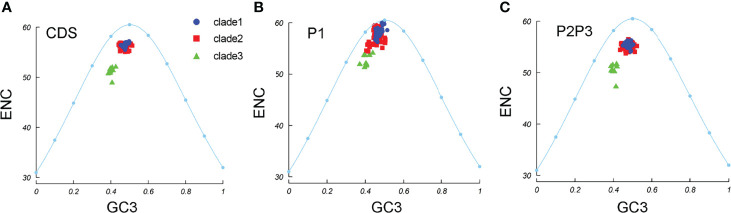
ENC-GC3 plots. The curve indicates the expected codon usage if GC compositional constraints alone account for the codon usage bias. **(A)** coding sequences, **(B)** P1 region, **(C)** P2P3 region. The blue dots, red squares, and green triangles indicate clade 1, clade 2 and clade 3 strains.

To understand whether pressure dominates in the *EV-A* codon usage bias, ENC–GC3 plots were constructed. We observed that all the *EV-A* strains clustered together under the expected ENC curve (**Figure 6A**). This suggests that natural selection may affect the genomic evolution of *EV-A* strains. The *EV-A* clades deviated from the expected curve differently and the clade 3 strains were lower below the curve, suggesting that clade 3 strains are subjected to higher selective pressure than other clades. The clade-specific difference can also be seen in P1 and P2P3 coding regions (**Figures 6B, C**). However, the effects of mutation pressure and natural selection on individual proteins varied (**Supplementary Figure S3**). All 2BC and 3D gene data points fell below the expected curve, while the 3AB, 3C, VP3 and VP4-VP2 coding sequences of some strains were aggregated on the curve, suggesting a dominant influence due to mutation pressure. It is noteworthy that clade 3 strains are the lower points under the curve in 3AB, 3D and VP3 plots, indicating a greater effect by natural selection in these coding regions.

### 3.7 Natural Selection Predominates in Shaping the Codon Usage Patterns in EV-A

**Figure 7:**
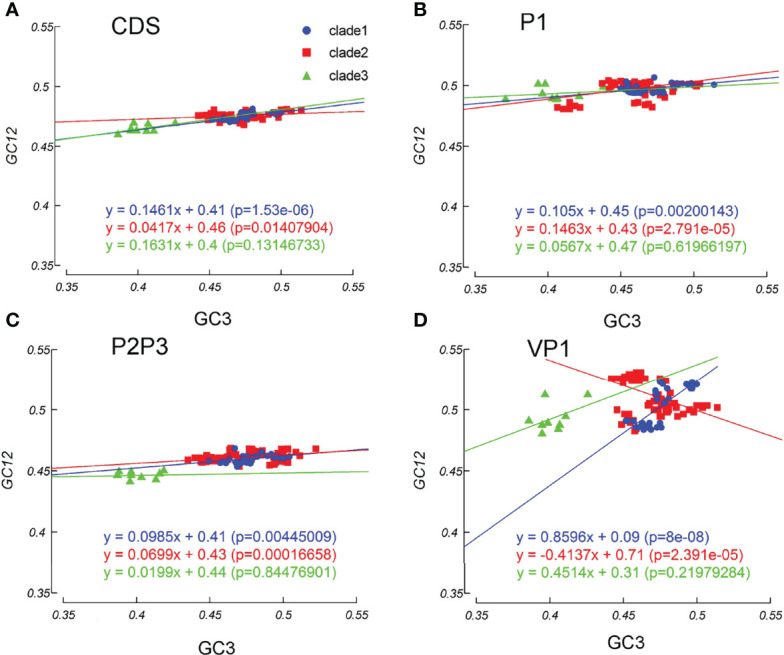
Neutrality plot analysis. Neutrality plots (GC12 and GC3) was constructed for the coding sequences, P1 and P2P3 regions of *EV-A* strains. **(A)** coding sequences, **(B)** P1 region, **(C)** P2P3 region, **(D)** VP1 gene. The blue dots, red squares, and green triangles indicate clade 1, clade 2 and clade 3 strains.

Mutation pressure and natural selection help to shape the codon usage patterns. To study the magnitude of each force, we used the neutrality plot analysis. We observed a significant positive correlation between GC12 and GC3 values across the *EV-A* coding sequences (slope =0.113, r =0.695, p=0.007). The linear regression slope suggested that the relative neutrality (mutation pressure) was 11.31%, and the relative constraint on GC3 (natural selection) was 89.69%, indicating that the *EV-A* codon usage patterns were primarily shaped by natural selection. Regression slopes in clade 1 and clade 2 are 0.1461 and 0.0417, while the correlation is not significant in clade 3, consistently indicating high levels of influence on codon patterns by natural selection (**Figure 7A**). The results of the neutrality plot analysis in P1 and P2P3 regions were similar to the whole coding sequences (**Figures 7B, C**). We analyzed individual proteins in three clades, and observed significant correlations between the GC12 and GC3 values of the VP1, 2A, 3D sequences in clade 1 and the VP3, 2BC, 3D coding sequences in clade 2 strains. All slope values for each protein are below 0.5 (from 0.007 to 0.4076), which supports the stronger effects of selection pressure than mutation pressure (**Supplementary Figure S4**). Notably, the VP1 coding sequences in clade 1 showed an exceptional slope value= 0.8696, indicating a weak selection force in this region (**Figure 7D**). Overall, our data suggest that the influence of natural selection prevails over the coding sequences of *EV-A* strains.

### 3.8 EV-A Showed Host-Specific Codon Adaptation Patterns

**Figure 8:**
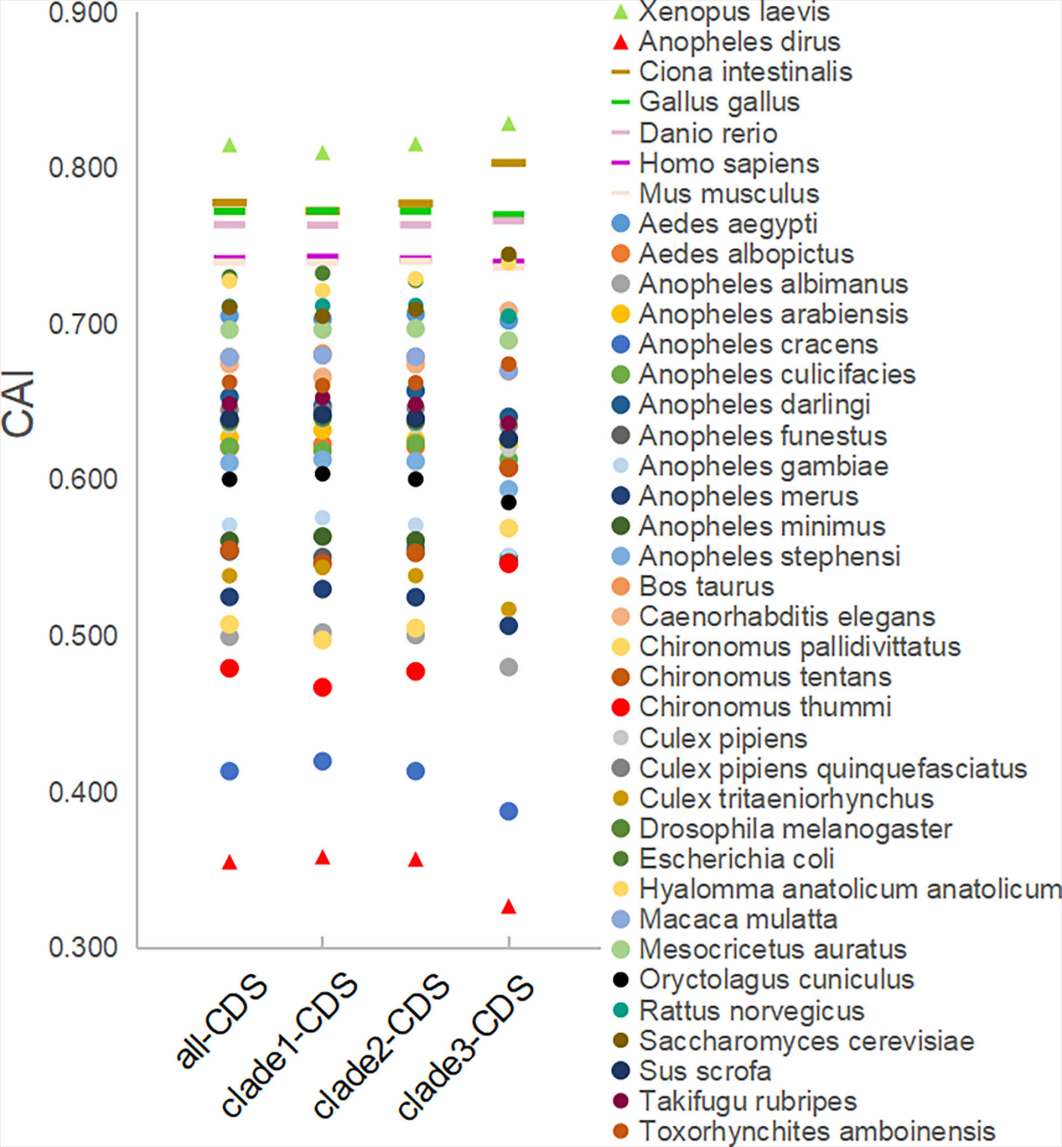
CAI analysis of the *EV-A* coding sequences against model organisms. CDS stands for the coding sequences of *EV-A* strains.

The CAI analysis reflects the fitness of the viral gene for the host cell. The CAI values were calculated for each coding sequence against 38 model organisms based on the CUTG database. The six highest average CAI values obtained for the entire *EV-A* coding sequence were assigned to *Xenopus laevis* (0.82 ± 0.006), *Ciona intestinalis* (0.78 ± 0.009), *Gallus gallus* (0.77 ± 0.005), *Danio rerio* (0.76 ± 0.005), *Homo sapiens* (0.74 ± 0.005), and *Mus musculus* (0.74 ± 0.005) (**Figure 8** and **Supplementary Table S4A**). For all six matched species, the CAI analyses of the P1 and P2P3 regions are consistent with the entire coding sequence. We also observed that the CAI values of clade 3 is significantly higher than clade 1 and clade 2 in *Xenopus laevis and Ciona intestinalis* (p<0.01), indicating that *EV-A* strains show clade-specific codon adaptation patterns.

In addition, we also calculate the RCDI and SiD values to show the degree of unsuitability and potential impact of model organism codon usage patterns on *EV-A*. (**Supplementary Figure S5**, and **Supplementary Table S4**). The six lowest average RCDI values of the entire EV-A coding sequence include *Xenopus laevis* (1.06), *Ciona interstinals* (1.09), *Gallus gallus* (1.11), *Danio rerio* (1.11), *Mus musculus* (1.12), *Homo sapiens* (1.12). Among the 38 model animals, the six species with the lowest mean SiD values also matched the CAI and RCDI analysis, indicating that they have similar codon bias patterns and may be most appropriate hosts for *EV-A*. The data of the P1 and P2P3 regions are consistent with the entire coding sequence.

### 3.9 Specific Codon Bias can Affect the Protein Expression Level of EV-A

**Figure 9:**
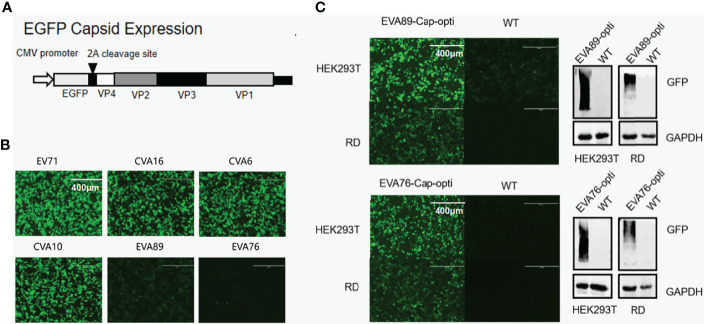
Codon optimization and protein expression levels for *EV-A* strains. **(A)** Schematic map of P1 protein expression plasmid. **(B)** Post plasmid transfection, GFP-tagged P1 protein levels of different *EV-A* strains in HEK293T cells was monitored by fluorescence microscopy. **(C)** Comparison of EVA76 and EVA89 P1 protein levels before and after codon optimization. Expression of GFP-tagged P1 protein in the cell culture was monitored through fluorescence microscopy (left panel). Furthermore, the expression of GFP-tagged P1 proteins was examined by anti-GFP antibodies using Western Blot (right panel). Housekeeping gene GAPDH was used for loading control.

As above, we concluded that there are significant differences in codon bias patterns between conventional and unconventional *EV-A* strains. To explore whether the codon bias can affect the protein translation of *EV-A*, we first tested if there are differences in protein translation between viruses representative of three clades. The expression of GFP-tagged viral capsid protein in HEK293T cells was examined by fluorescence microscopy (**Figures 9A, B**). It was found that EV71 and CVA16 of clade 1, CVA6 and CVA10 of clade 2 had similar levels of expression, significantly higher than EVA76 and EVA89 of clade 3. This result indicates that the expression levels of conventional *EV-A capsids* are substantially higher than those of unconventional *EV-A*.

To confirm if the lower expression levels of unconventional clades are caused by codon bias, we have optimized the codon of the capsid region of EVA89 and EVA76 according to *Homo sapiens*. The optimized fragments EVA76-Cap-opti and EVA89-Cap-opti were expressed in HEK293T and RD cell lines. Using immunofluorescence microscopy and Western blot, we found that the optimized capsid protein of EVA89 and EVA76 expressed at significantly higher levels (**Figure 9C**), showing that the codon bias of clade 3 does contribute to the lower protein expression.

## 4 Discussion

This study analyzed the codon usage bias in the *EV-A* in order to understand its evolutionary patterns. It is known that recombination events can affect the codon usage patterns and the phylogenetic tree topology, which can lead to misinterpretations. Therefore, we excluded all possible recombinants and the remaining 125 complete *EV-A* genomes were analyzed. Based on phylogenetic analysis, *EV-A* is divided into three clades: clade 1, clade 2 and clade 3. The clade 1 comprises CVA7, CVA14, CVA16, EV71 and EV120, of which EV71 and CVA16 are principle pathogens of HFMD. Clade 2 includes CVA2, CVA3, CVA4, CVA5, CVA6, CVA8, CVA10 and CVA12, of which CVA6 and CVA10 are emerging recently. Clade 3 includes the unconventional strains, EVA76, 89, 90, 91 and 92.

Mutational pressure and natural selection are known to account for the codon usage bias, which has been reported in many species, including RNA virus. Here we use multiple methods to explore the codon bias of the *EV-A* strains and quantify the effect of mutation pressure and natural selection. First, ENC values of *EV-A* show a low overall codon usage bias, consistent with many RNA viruses (Jenkins and Holmes, 2003). Here we analyzed the RSCU pattern of *EV-A*, and found that *EV-A* evolved a mixed pattern of coincident and antagonistic codons relative to humans. This suggests that *EV-A* may have a complicated process of adapting to humans as host. When the codon usage between the virus and the host is consistent, the host can more effectively translate the corresponding virus amino acid codons and produce proteins faster, where the virus must compete with its host. In contrast, the use of antagonistic codons can avoid competition with the host and the virus protein can be folded better to get a more stable viral capsid, although this may reduce the translation effectiveness of the viral amino acid codons. Previous studies indicate that the codon bias pattern of poliovirus is very similar to that of humans, whereas the hepatitis A virus and EB virus have developed a codon usage pattern which is largely antagonistic to the host (Karlin et al., 1990; Pintó et al., 2018). Thus, different codon adaptation patterns may be related to the translation shutdown mechanism, virus life cycle and transmission pathway.

The codon usage bias may be strongly influenced by the overall nucleotide composition. For viruses, the GC- or AU-rich compositions tend to correlate with their RSCU patterns. In addition, GC- or AU-rich genomes tend to contain codons preferentially ending with either G/C or A/U, respectively. Such trends, if present, support the influence of mutation pressure. Here, we observed that *EV-A* genomes are AU-rich and prefer to end with A/U, which supports a mutation pressure effect. We also find a proportion of CpG and UpA dinucleotide in *EV-A* as seen in nearly all RNA viruses (Karlin et al., 1994), that is probably linked to evasion of antiviral immune responses (Sugiyama et al., 2005; Greenbaum et al., 2009). Microbial DNA sequences containing unmethylated CpG dinucleotides can trigger an immune response through the Toll-like receptor 9 (Latz et al., 2004). A similar sensor for CpG in RNA viruses was likely present, but it remains to be discovered (Greenbaum et al., 2009; Atkinson et al., 2014), while UpA is susceptible to cleavage by RNaseL (Steil and Barton, 2009). The importance of dinucleotide compositions has been demonstrated in attenuation of RNA virus by codon deoptimization after artificial increase of CpG/UpA (Atkinson et al., 2014).

The findings from ENC and nucleotide composition analysis suggest that both mutation and selection pressure may shape the *EV-A* genome. Then, by the neutrality plot, we conclude that the selection pressure is dominant in *EV-A* codon bias, due to the non-significant correlation or near-zero slope between the GC12 and GC3 values. This finding is not consistent with a previous EV71 report (Ma et al., 2014), probably because previous studies based only on nucleotide composition and the ENC-GC3 plot, which is inadequate (Wright, 1990). In contrast, we conducted neutrality plot analysis which provided a more robust support to the conclusion.

We also analyzed each protein sequence by ENC-GC3 plot and neutrality plot. It is worth noting that the effects of both forces on each protein are different. In the neutrality plots, natural selection is found to be the dominant factor in the formation of codon usage patterns. However, VP1 in clade 1 is mainly affected by mutation pressure, rather than by natural selection. For specific proteins of different strains, their ENC-GC3 plot is not always consistent. Some protein sequences of particular *EV-A* strains are distributed on the curve, indicating that they are primarily affected by mutation pressure.

In the COA analysis, clade 1 and clade 2 strains clustered together while clade 3 is in a separate cluster, suggesting that clade 3 strains have different codon usage patterns with clade 1 and clade 2. However, at the individual gene level, many clusters of all three clades overlap, indicating that *EV-A* strains likely originate from common ancestors and have undergone subsequent evolutionary divergence, especially for 3C, 3D and VP3. We also noticed that clustering is not entirely consistent in the COA plots with phylogenetic analysis. Clade 1 and clade 2 aggregate to form cluster 1, while CVA5 and CVA6 in clade 2 form cluster 2 separately, likely due to phylogenetic analysis algorithms ignoring differences in nucleotide differences at the 3rd codon position. These results indicate that CVA5 and CVA6 differ from other clade 2 viruses in codon usage patterns, in particular the 3rd codon position, whereas other clade 2 viruses have similar codon usage patterns with clade 1. Based on the RSCU, ENC and COA analyses, we also found that there were differences in codon bias between *EV-A* P1 and P2-P3 regions. The codon bias of P2-P3 region is higher than that of P1 region, and is subjected to more selective pressure and tends to use U and C more frequently.

The CAI, RCDI and SiD analyses suggest that the suitable host species in terms of codon adaptability for *EV-A* may include: *Xenopus laevis*, *Ciona intestinalis*, *Gallus gallus*, *Danio rerio*, *Homo sapiens* and *Mus musculus*, whereas the more suitable hosts for the clade 3 viruses are likely to be *Xenopus laevis*, *Ciona interstinals.* Given its unique codon bias, it is tempting to assume that the clade 3 viruses may circulate in different host species other than *EV-A* clade 1 and clade 2 viruses. However, human and non-human primates are the only known natural hosts for *EV-A (* Wang and Yu, 2014 *;* Mombo et al., 2017 *)*. In previous surveys, clade 3 viruses were also detected in mandrill, chimpanzee, rhesus macaque, baboon (Oberste et al., 2013). In the future, the verification at the cellular or animal level is required to determine whether the analyzed model organisms are indeed possible hosts for *EV-A*. Despite enterovirus have narrow host ranges, their SiD values are relatively low and similar to virus with broad host ranges. Otherwise, this may suggest that enterovirus do not need high codon compatibility with its host to replicate rapidly (Tian et al., 2018).

Clade 3 viruses have a unique phylogenetic relationship, low pathogenicity and epidemic features with respect to other *EV-A* strains (Fan et al., 2015). Here, we have found a unique codon usage pattern in clade 3 strains which may explain these differences. Indeed, our experiments have shown that codon optimization of the clade 3-P1 constructs increases their expressions to comparable levels to other clades. Following codon optimization, the CAI value of EVA76 over *homo sapiens* was also raised from 0.74 to 0.84. Since P2P3 regions have a higher codon bias and different function with P1 region, it is possible that optimization on P2P3 regions also improves the virus activity in human cell lines. Curiously, the codon deoptimization of conventional enteroviruses has made them less pathogenic and vaccine candidates (Tsai et al., 2019; Lee et al., 2021), one waits to see if codon optimization will now make clade 3 enteroviruses more virulent.

With low replication fidelity and frequent recombination, enteroviruses exhibit high genetic diversity and a potential for cross-species infection. Studies of *EV-A* infection in non-human primates provide increasing evidence of a risk of zoonotic transmission between animals and humans (Mombo et al., 2017). In fact, the pathogenic spectrum of *EV-A* is evolving rapidly, from EV71 and CVA16 previously to CVA10 and CVA6, which have caused many epidemics in recent years. It is unclear if the unconventional *EV-A* strains will cause epidemics over time, although we have recently reported that viable recombinants between conventional and unconventional *EV-A* types could be generated successfully (Wang et al., 2020). Thus, it is of great interest to study the evolutionary mechanism of viruses, especially these forthcoming unconventional viruses.

## 5 Conclusions

Our study shows that *EV-A* has developed clade-specific codon bias patterns. Both mutation pressure and natural selection affected the codon usage patterns in *EV-A*. These data have new implications for vaccine development and management of *EV-A* infection.

## Data Availability Statement

The original contributions presented in the study are included in the article/**Supplementary Material**. Further inquiries can be directed to the corresponding authors.

## Author Contributions

Conceptualization, LiYZ, JQX, XYZ and SYZ. Data curation, LiYZ and MC. Formal analysis, LiYZ and MC. Funding acquisition, LiYZ, SYZ and JJY. Investigation, LiYZ, MC, JJY and MW. Methodology, LiYZ and SYZ. Resources, JQX, XYZ and SYZ. Visualization, LiYZ, MC and LiuYZ. Writing—original draft preparation, LiYZ and MC. Writing—review and editing, LiYZ, JQX, XYZ and SYZ. Supervision, SYZ, JQX and XYZ. Project administration, LiYZ. and SYZ. All authors contributed to the article and approved the submitted version.

## Funding

This research was supported by the Fundamental Research Funds for the Shanghai Public Health Clinical Center (grant number: KY-GW-2016-04 and KY-GW-2018-17) and National Natural Science Foundation of China (82002135).

## Conflict of Interest

The authors declare that the research was conducted in the absence of any commercial or financial relationships that could be construed as a potential conflict of interest.

## Publisher’s Note

All claims expressed in this article are solely those of the authors and do not necessarily represent those of their affiliated organizations, or those of the publisher, the editors and the reviewers. Any product that may be evaluated in this article, or claim that may be made by its manufacturer, is not guaranteed or endorsed by the publisher.

## Supplementary Material

Supplementary Figure 1 Phylogenetic analysis of the *EV-A* stains.

Supplementary Figure 2 Genotype-specific of correspondence analysis plots were constructed for individual *EV-A* coding sequences.

Supplementary Figure 3 ENC–GC3 plots.

Supplementary Figure 4 Neutrality plot analysis.

Supplementary Figure 5 RCDI and SiD analysis of the EV-A coding sequences against model organisms.

Supplementary Table 1 Demographics of *Enterovirus A* genomes analyzed in present study.

Supplementary Table 2 Nucleotide composition analysis of *EV-A* coding sequences.

Supplementary Table 3 One way Anova analysis between three clades.

Supplementary Table 4 Summary data of CAI, RCDI and SiD analysis.

## References

[B1] Atkinson N. J. Jeroen W. Evans D. J. Peter S. (2014). The Influence of CpG and UpA Dinucleotide Frequencies on RNA Virus Replication and Characterization of the Innate Cellular Pathways Underlying Virus Attenuation and Enhanced Replication. Nucleic Acids Res 42 (7), 4527–4545. doi: 10.1093/nar/gku075 24470146 PMC3985648

[B2] Belalov I. S. Lukashev A. N. (2013). Causes and Implications of Codon Usage Bias in RNA Viruses. PLoS One 8 (2), e56642. doi: 10.1371/journal.pone.0056642 23451064 PMC3581513

[B3] Bian L. Wang Y. Yao X. Mao Q. Xu M. Liang Z. (2015). Coxsackievirus A6: A New Emerging Pathogen Causing Hand, Foot and Mouth Disease Outbreaks Worldwide. Expert Rev. Anti-Infect. Ther. 13 (9), 1061–1071. doi: 10.1586/14787210.2015.1058156 26112307

[B4] Blomqvist S. Klemola P. Kaijalainen S. Paananen A. Simonen M.-L. Vuorinen T.. (2010). Co-Circulation of Coxsackieviruses A6 and A10 in Hand, Foot and Mouth Disease Outbreak in Finland. J. Clin. Virol. 48 (1), 49–54. doi: 10.1016/j.jcv.2010.02.002 20189452

[B5] Bulmer M. (1991). The Selection-Mutation-Drift Theory of Synonymous Codon Usage. Genetics 129 (3), 897–907. doi: 10.1093/genetics/129.3.897 1752426 PMC1204756

[B6] Chen P. Song Z. Qi Y. Feng X. Xu N. Sun Y.. (2012). Molecular Determinants of Enterovirus 71 Viral Entry Cleft Around Gln-172 on VP1 Protein Interacts With Variable Region on Scavenge Receptor B 2. J. Biol. Chem. 287 (9), 6406–6420. doi: 10.1074/jbc.M111.301622 22219187 PMC3307280

[B7] Comeron J. M. Aguadé M. (1998). An Evaluation of Measures of Synonymous Codon Usage Bias. J. Mol. Evol. 47 (3), 268–274. doi: 10.1007/PL00006384 9732453

[B8] Fan Q. Zhang Y. Hu L. Sun Q. Cui H. Yan D.. (2015). A Novel Recombinant Enterovirus Type EV-A89 With Low Epidemic Strength in Xinjiang, China. Sci. Rep. 5, 18558. doi: 10.1038/srep18558 26685900 PMC4685259

[B9] Friendly M. (2002). Corrgrams: Exploratory Displays for Correlation Matrices. Am. Stat. 56 (4), 316–324. doi: 10.1198/000313002533

[B10] Grantham R. Gautier C. Gouy M. (1980). Codon Frequencies in 119 Individual Genes Confirm Corsistent Choices of Degenerate Bases According to Genome Type. Nucleic Acids Res. 8 (9), 1893–1912. doi: 10.1093/nar/8.9.1893 6159596 PMC324046

[B11] Greenbaum B. D. Rabadan R. Levine A. J. (2009). Patterns of Oligonucleotide Sequences in Viral and Host Cell RNA Identify Mediators of the Host Innate Immune System. PloS One 4 (6), e5969. doi: 10.1371/journal.pone.0005969 19536338 PMC2694999

[B12] Jenkins G. M. Holmes E. C. (2003). The Extent of Codon Usage Bias in Human RNA Viruses and its Evolutionary Origin. Virus Res. 92 (1), 1–7. doi: 10.1016/S0168-1702(02)00309-X 12606071

[B13] Karlin S. Blaisdell B. E. Schachtel G. A. (1990). Contrasts in Codon Usage of Latent Versus Productive Genes of Epstein-Barr Virus: Data and Hypotheses. J. Virol. 64 (9), 4264–4273. doi: 10.1128/jvi.64.9.4264-4273.1990 2166815 PMC247892

[B14] Karlin S. Doerfler W. Cardon L. (1994). Why is CpG Suppressed in the Genomes of Virtually All Small Eukaryotic Viruses But Not in Those of Large Eukaryotic Viruses? J. Virol. 68 (5), 2889–2897. doi: 10.1128/jvi.68.5.2889-2897.1994 8151759 PMC236777

[B15] Karlin S. Mrazek J. Campbell A. M. (1997). Compositional Biases of Bacterial Genomes and Evolutionary Implications. J. Bacteriol. 179 (12), 3899–3913. doi: 10.1128/jb.179.12.3899-3913.1997 9190805 PMC179198

[B16] Kumar S. Stecher G. Tamura K. (2016). MEGA7: Molecular Evolutionary Genetics Analysis Version 7.0 for Bigger Datasets. Mol. Biol. Evol. 33 (7), 1870–1874. doi: 10.1093/molbev/msw054 27004904 PMC8210823

[B17] Latz E. Schoenemeyer A. Visintin A. Fitzgerald K. A. Monks B. G. Knetter C. F.. (2004). TLR9 Signals After Translocating From the ER to CpG DNA in the Lysosome. Nat. Immunol. 5 (2), 190–198. doi: 10.1038/ni1028 14716310

[B18] Lee M. H. P. Tan C. W. Tee H. K. Ong K. C. Sam I. C. Chan Y. F. (2021). Vaccine Candidates Generated by Codon and Codon Pair Deoptimization of Enterovirus A71 Protect Against Lethal Challenge in Mice. Vaccine 39 (12), 1708–1720. doi: 10.1016/j.vaccine.2021.02.024 33640144

[B19] Liu W. Wu S. Xiong Y. Li T. Wen Z. Yan M.. (2014). Co-Circulation and Genomic Recombination of Coxsackievirus A16 and Enterovirus 71 During a Large Outbreak of Hand, Foot, and Mouth Disease in Central China. PloS One 9 (4), e96051. doi: 10.1371/journal.pone.0096051 24776922 PMC4002479

[B20] Liu Y.-S. Zhou J.-H. Chen H.-T. Ma L.-N. Pejsak Z. Ding Y.-Z.. (2011). The Characteristics of the Synonymous Codon Usage in Enterovirus 71 Virus and the Effects of Host on the Virus in Codon Usage Pattern. Infect. Genet. Evol. 11 (5), 1168–1173. doi: 10.1016/j.meegid.2011.02.018 21382519 PMC7185409

[B21] Lukashev A. N. Shumilina E. Y. Belalov I. S. Ivanova O. E. Eremeeva T. P. Reznik V. I.. (2014). Recombination Strategies and Evolutionary Dynamics of the Human Enterovirus A Global Gene Pool. J. Gen. Virol 95, 868–873. doi: 10.1099/vir.0.060004-0 24425417

[B22] Ma M. Hui L. Wang M. Tang Y. Chang Y. Jia Q. (2014). Overall Codon Usage Pattern of Enterovirus 71. Genet. Mol. Res. 13 (1), 336–343. doi: 10.4238/2014.January.21.1 24535860

[B23] Mao Q. Wang Y. Yao X. Bian L. Wu X. Xu M.. (2014). Coxsackievirus A16: Epidemiology, Diagnosis, and Vaccine. Hum. Vaccines Immunother. 10 (2), 360–367. doi: 10.4161/hv.27087 PMC4185891 24231751

[B24] Martin D. P. Murrell B. Golden M. Khoosal A. Muhire B. (2015). RDP4: Detection and Analysis of Recombination Patterns in Virus Genomes. Virus Evol. 1 (1), vev003. doi: 10.1093/ve/vev003 27774277 PMC5014473

[B25] Mombo I. M. Lukashev A. N. Bleicker T. Brünink S. Berthet N. Maganga G. D.. (2017). African non-Human Primates Host Diverse Enteroviruses. PLoS One 12 (1), e0169067. doi: 10.1371/journal.pone.0169067 28081564 PMC5233426

[B26] Mueller S. Papamichail D. Coleman J. R. Skiena S. Wimmer E. (2006). Reduction of the Rate of Poliovirus Protein Synthesis Through Large-Scale Codon Deoptimization Causes Attenuation of Viral Virulence by Lowering Specific Infectivity. J. Virol. 80 (19), 9687–9696. doi: 10.1128/JVI.00738-06 16973573 PMC1617239

[B27] Nakamura Y. Gojobori T. Ikemura T. (2000). Codon Usage Tabulated From International DNA Sequence Databases: Status for the Year 2000. Nucleic Acids Res. 28 (1), 292–292. doi: 10.1093/nar/28.1.292 10592250 PMC102460

[B28] Oberste M. S. Feeroz M. M. Maher K. Nix W. A. Engel G. A. Hasan K. M.. (2013). Characterizing the Picornavirus Landscape Among Synanthropic Nonhuman Primates in Bangladesh 2007 to 2008. J. Virol. 87 (1), 558–571. doi: 10.1128/JVI.00837-12 23097448 PMC3536389

[B29] Oberste M. S. Gerber S. I. (2014). “Enteroviruses and Parechoviruses: Echoviruses, Coxsackieviruses, and Others” In:Kaslow R. Stanberry L. Le Duc J.(eds) Viral Infections of Humans (Springer, Boston, MA) 225–252. doi: 10.1007/978-1-4899-7448-8_11

[B30] Oberste M. S. Maher K. Michele S. M. Belliot G. Uddin M. Pallansch M. A. (2005). Enteroviruses 76, 89, 90 and 91 Represent a Novel Group Within the Species Human Enterovirus A. J. Gen. Virol. 86 (2), 445–451. doi: 10.1099/vir.0.80475-0 15659764

[B31] Organization, W.H (2011). "A Guide to Clinical Management and Public Health Response for Hand, Foot and Mouth Disease (HFMD)" (Manila: WHO Regional Office for the Western Pacific).

[B32] Peden J. F. (1999). Analysis of Codon Usage. (University of Nottingham, United Kingdom). doi: 10.1006/expr.1997.4185. Available at: https://sourceforge.net/projects/codonw/.

[B33] Pickett B. E. Sadat E. L. Zhang Y. Noronha J. M. Squires R. B. Hunt V.. (2012). ViPR: An Open Bioinformatics Database and Analysis Resource for Virology Research. Nucleic Acids Res. 40 (D1), D593–D598. doi: 10.1093/nar/gkr859 22006842 PMC3245011

[B34] Pintó R. M. Pérez-Rodríguez F.-J. D’Andrea L. de Castellarnau M. Guix S. Bosch A. (2018). Hepatitis A Virus Codon Usage: Implications for Translation Kinetics and Capsid Folding. Cold Spring Harbor Perspect. Med. 8 (10), a031781. doi: 10.1101/cshperspect.a031781 PMC6169985 29530949

[B35] Puigbò P. Bravo I. G. Garcia-Vallve S. (2008). CAIcal: A Combined Set of Tools to Assess Codon Usage Adaptation. Biol. Direct. 3 (1), 1–8. doi: 10.1186/1745-6150-3-38 18796141 PMC2553769

[B36] Sharp P. M. Averof M. Lloyd A. T. Matassi G. Peden J. F. (1995). DNA Sequence Evolution: The Sounds of Silence. Philos. Trans. R. Soc. London. Ser. B.: Biol. Sci. 349 (1329), 241–247. doi: 10.1098/rstb.1995.0108 8577834

[B37] Sharp P. M. Li W.-H. (1986). Codon Usage in Regulatory Genes in Escherichia Coli Does Not Reflect Selection for ‘Rare’codons. Nucleic Acids Res. 14 (19), 7737–7749. doi: 10.1093/nar/14.19.7737 3534792 PMC311793

[B38] Sharp P. M. Li W.-H. (1987). The Codon Adaptation Index-a Measure of Directional Synonymous Codon Usage Bias, and its Potential Applications. Nucleic Acids Res. 15 (3), 1281–1295. doi: 10.1093/nar/15.3.1281 3547335 PMC340524

[B39] Simmonds P. Tuplin A. Evans D. J. (2004). Detection of Genome-Scale Ordered RNA Structure (GORS) in Genomes of Positive-Stranded RNA Viruses: Implications for Virus Evolution and Host Persistence. Rna 10 (9), 1337–1351. doi: 10.1261/rna.7640104 15273323 PMC1370621

[B40] Staring J. van den Hengel L. G. Raaben M. Blomen V. A. Carette J. E. Brummelkamp T. R. (2018). KREMEN1 Is a Host Entry Receptor for a Major Group of Enteroviruses. Cell Host Microbe 23 (5), 636–643.e635. doi: 10.1016/j.chom.2018.03.019 29681460

[B41] Steil B. P. Barton D. J. (2009). Cis-Active RNA Elements (CREs) and Picornavirus RNA Replication. Virus Res. 139 (2), 240–252. doi: 10.1016/j.virusres.2008.07.027 18773930 PMC2692539

[B42] Sueoka N. (1988). Directional Mutation Pressure and Neutral Molecular Evolution. Proc. Natl. Acad. Sci. 85 (8), 2653–2657. doi: 10.1073/pnas.85.8.2653 3357886 PMC280056

[B43] Sueoka N. (1995). Intrastrand Parity Rules of DNA Base Composition and Usage Biases of Synonymous Codons. J. Mol. Evol. 40 (3), 318–325. doi: 10.1007/BF00163236 7723058

[B44] Sueoka N. (1999). Translation-Coupled Violation of Parity Rule 2 in Human Genes is Not the Cause of Heterogeneity of the DNA G+ C Content of Third Codon Position. Gene 238 (1), 53–58. doi: 10.1016/S0378-1119(99)00320-0 10570983

[B45] Sugiyama T. Gursel M. Takeshita F. Coban C. Conover J. Kaisho T.. (2005). CpG RNA: Identification of Novel Single-Stranded RNA That Stimulates Human CD14+ CD11c+ Monocytes. J. Immunol. 174 (4), 2273–2279. doi: 10.4049/jimmunol.174.4.2273 15699162

[B46] Su W. Li X. Chen M. Dai W. Sun S. Wang S.. (2017). Synonymous Codon Usage Analysis of Hand, Foot and Mouth Disease Viruses: A Comparative Study on Coxsackievirus A6, A10, A16, and Enterovirus 71 From 2008 to 2015. Infect. Genet. Evol.: J. Mol. Epidemiol. Evol. Genet. Infect. Dis. 53, 212–217. doi: 10.1016/j.meegid.2017.06.004 28602802

[B47] Tao Z. Cui N. Xu A. Liu Y. Song L. Li Y.. (2013). Isolation and Genomic Characterization of Three Enterovirus 90 Strains in Shandong, China. Arch. Virol. 158 (2), 479–483. doi: 10.1007/s00705-012-1517-2 23081679

[B48] Tian L. Shen X. Murphy R. W. Shen Y. (2018). The Adaptation of Codon Usage of+ ssRNA Viruses to Their Hosts. Infect. Genet. Evol. 63, 175–179. doi: 10.1016/j.meegid.2018.05.034 29864509 PMC7106036

[B49] Tsai Y. H. Huang S. W. Hsieh W. S. Cheng C. K. Chang C. F. Wang Y. F.. (2019). Enterovirus A71 Containing Codon-Deoptimized VP1 and High-Fidelity Polymerase as Next-Generation Vaccine Candidate. J. Virol. 93 (13), e02308–18. doi: 10.1128/JVI.02308-18 30996087 PMC6580961

[B50] Wang S.-M. Liu C.-C. (2014). Update of Enterovirus 71 Infection: Epidemiology, Pathogenesis and Vaccine. Expert Rev. Anti-infect. Ther. 12 (4), 447–456. doi: 10.1586/14787210.2014.895666 24579906

[B51] Wang Y.-F. Yu C.-K. (2014). Animal Models of Enterovirus 71 Infection: Applications and Limitations. J. Biomed. Sci. 21 (1), 31. doi: 10.1186/1423-0127-21-31 24742252 PMC4013435

[B52] Wang M. Zhu L. Fan J. Yan J. Dun Y. Yu R.. (2020). Rules Governing Genetic Exchanges Among Viral Types From Different Enterovirus A Clusters. J. Gen. Virol. 101 (11), 1145–1155. doi: 10.1099/jgv.0.001479 32762804 PMC7879560

[B53] Wong E. H. Smith D. K. Rabadan R. Peiris M. Poon L. L. (2010). Codon Usage Bias and the Evolution of Influenza A Viruses. Codon Usage Biases of Influenza Virus. BMC Evol. Biol. 10 (1), 253. doi: 10.1186/1471-2148-10-253 20723216 PMC2933640

[B54] Wright F. (1990). The ‘Effective Number of Codons’ Used in a Gene. Gene 87 (1), 23–29. doi: 10.1016/0378-1119(90)90491-9 2110097

[B55] Xu A. Tao Z. Lin X. Liu Y. Zhang Y. Song L.. (2011). The Complete Genome Sequence of an Enterovirus 76 Isolate in China Reveals a Recombination Event. Arch. Virol. 156 (9), 1685–1689. doi: 10.1007/s00705-011-1067-z 21755310

[B56] Yamayoshi S. Yamashita Y. Li J. Hanagata N. Minowa T. Takemura T.. (2009). Scavenger Receptor B2 is a Cellular Receptor for Enterovirus 71. Nat. Med. 15 (7), 798–801. doi: 10.1038/nm.1992 19543282

[B57] Yip C. Lau S. Woo P. Yuen K. Y. (2013). Human Enterovirus 71 Epidemics: What's Next? Emerg. Health Threat. J. 6 (6), 19780. doi: 10.3402/ehtj.v6i0.19780 PMC3772321 24119538

[B58] Zhang H. Cao H.-W. Li F.-Q. Pan Z.-Y. Wu Z.-J. (2014). Analysis of Synonymous Codon Usage in Enterovirus 71. VirusDisease 25 (2), 243–248. doi: 10.1007/s13337-014-0215-y 25674591 PMC4188181

[B59] Zhang Z. Dong Z. Li J. Carr M. J. Zhuang D. Wang J.. (2017). Protective Efficacies of Formaldehyde-Inactivated Whole-Virus Vaccine and Antivirals in a Murine Model of Coxsackievirus A10 Infection. J. Virol. 91 (13), e00333–e00317. doi: 10.1128/JVI.00333-17 28424287 PMC5469256

[B60] Zhou J.-H. Zhang J. Sun D.-J. Ma Q. Chen H.-T. Ma L.-N.. (2013). The Distribution of Synonymous Codon Choice in the Translation Initiation Region of Dengue Virus. PLoS One 8 (10), e77239. doi: 10.1371/journal.pone.0077239 24204777 PMC3808402

[B61] Zhuang Z.-C. Kou Z.-Q. Bai Y.-J. Cong X. Wang L.-H. Li C.. (2015). Epidemiological Research on Hand, Foot, and Mouth Disease in Mainland China. Viruses 7 (12), 6400–6411. doi: 10.3390/v7122947 26690202 PMC4690870

