# Supplementary material for: Enterovirus A Shows Unique Patterns of Codon Usage Bias in Conventional Versus Unconventional Clade
Source: Front Cell Infect Microbiol. 2022 Jul 14;12:941325. doi: 10.3389/fcimb.2022.941325 (PMC9329520; doi:10.3389/fcimb.2022.941325)
Supplement: Supplementary Figure 1 — Phylogenetic analysis of the EV-A stains. [file DataSheet_1.pdf]

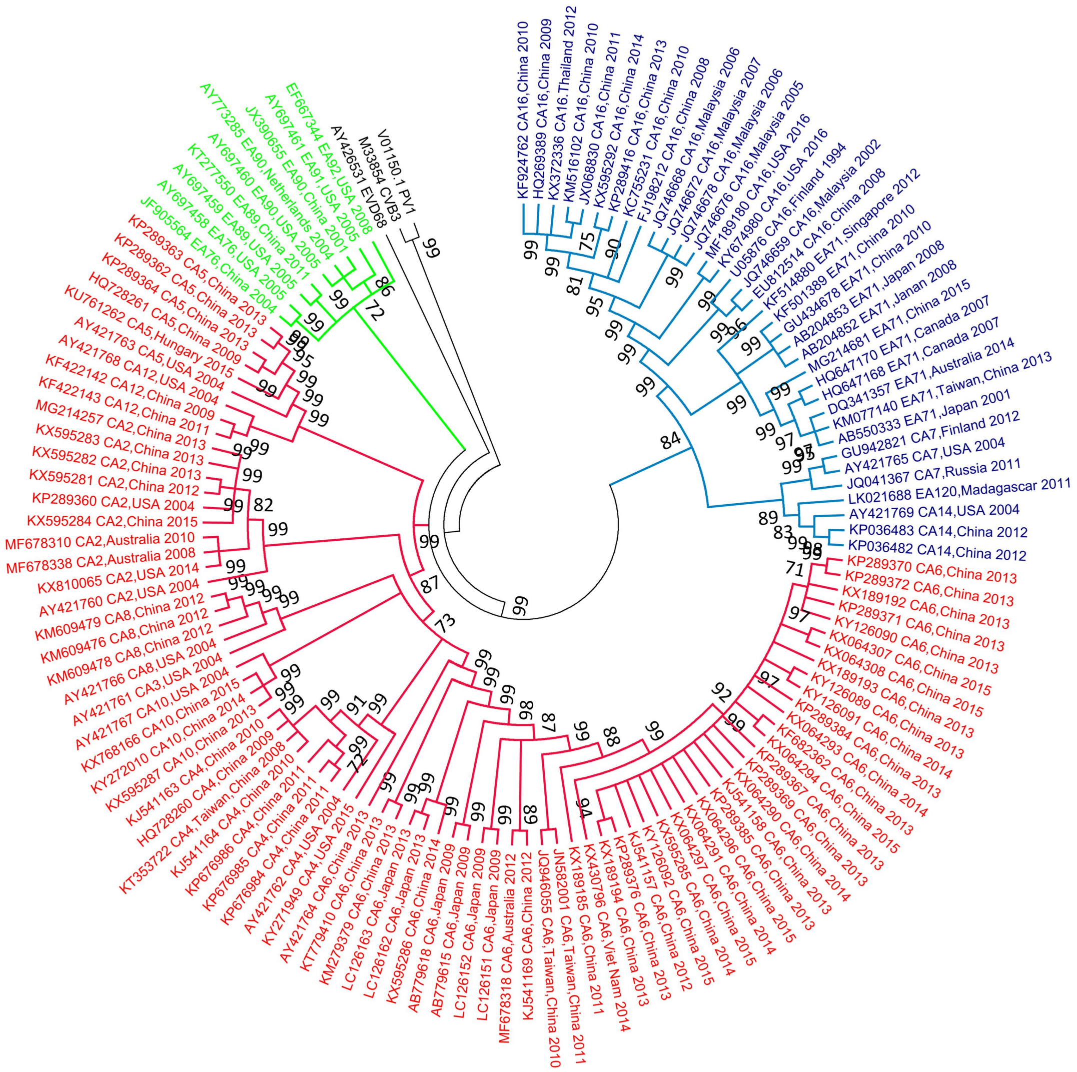

Supplementary Figure S1. Phylogenetic analysis of the *EV-A* strains

The maxmium likehood tree was constructed from the P1 region of 125 selected stains with the TN93+G model and 1000 bootstrap replicates. The blue, red and green subtrees are clade1,clade2 and clade3 respectively and the black subtrees is selected outer clade. Only strong bootstrap values (>70%) were shown.
