# Supplementary material for: Enterovirus A Shows Unique Patterns of Codon Usage Bias in Conventional Versus Unconventional Clade
Source: Front Cell Infect Microbiol. 2022 Jul 14;12:941325. doi: 10.3389/fcimb.2022.941325 (PMC9329520; doi:10.3389/fcimb.2022.941325)
Supplement: Supplementary Figure 2 — Genotype-specific of correspondence analysis plots were constructed for individual EV-A coding sequences. [file DataSheet_2.pdf]

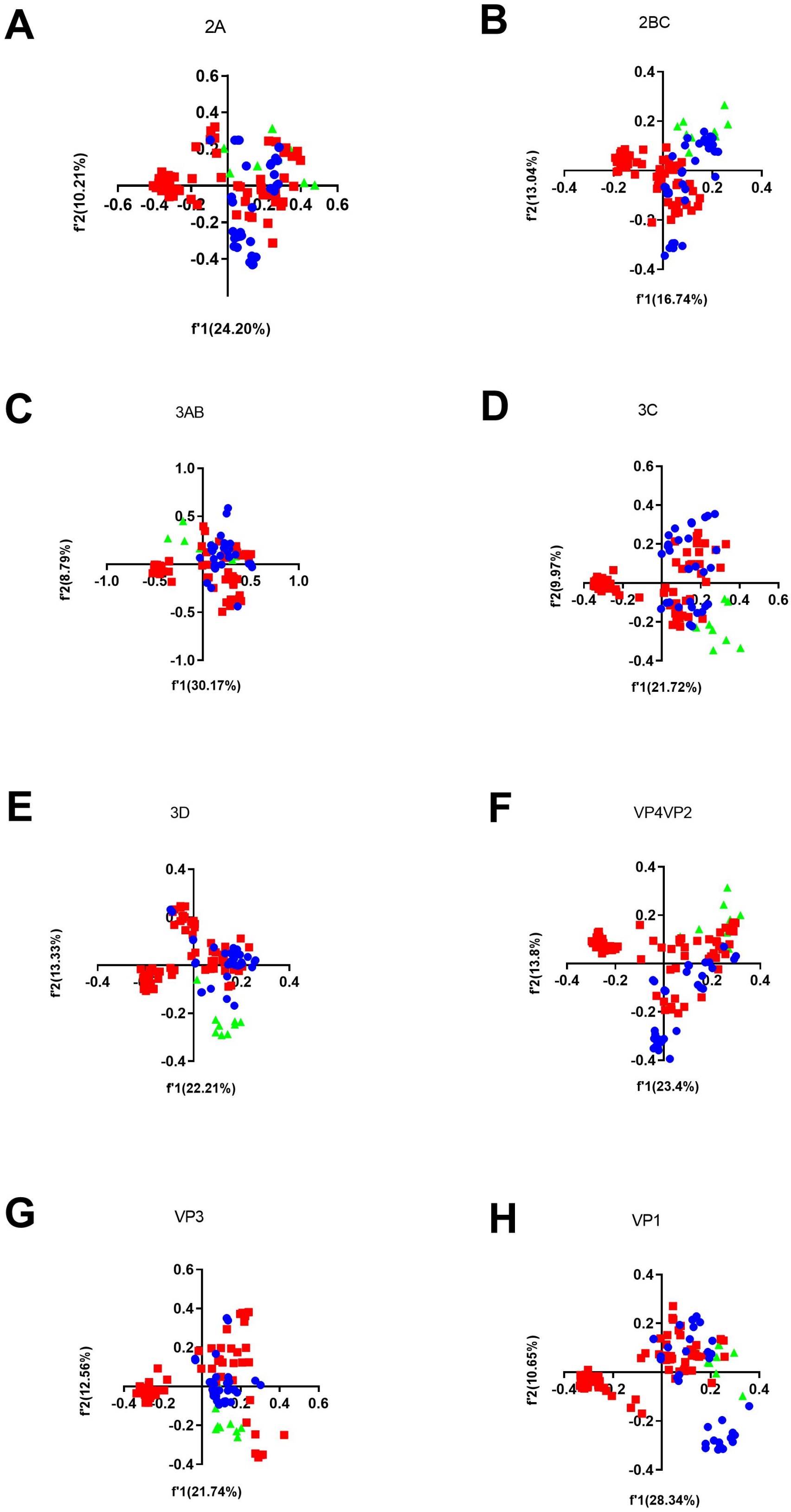

Supplementary Figure S2. Genotype-specific of correspondence analysis plots were constructed for individual *EV-A* coding sequences. (A) 2A, (B) 2BC, (C) 3AB, (D) 3C, (E) 3D, (F) VP4-VP2, (G) VP3 and (H) VP1.
