# Supplementary material for: Enterovirus A Shows Unique Patterns of Codon Usage Bias in Conventional Versus Unconventional Clade
Source: Front Cell Infect Microbiol. 2022 Jul 14;12:941325. doi: 10.3389/fcimb.2022.941325 (PMC9329520; doi:10.3389/fcimb.2022.941325)
Supplement: Supplementary Figure 3 — ENC–GC3 plots. [file DataSheet_3.pdf]

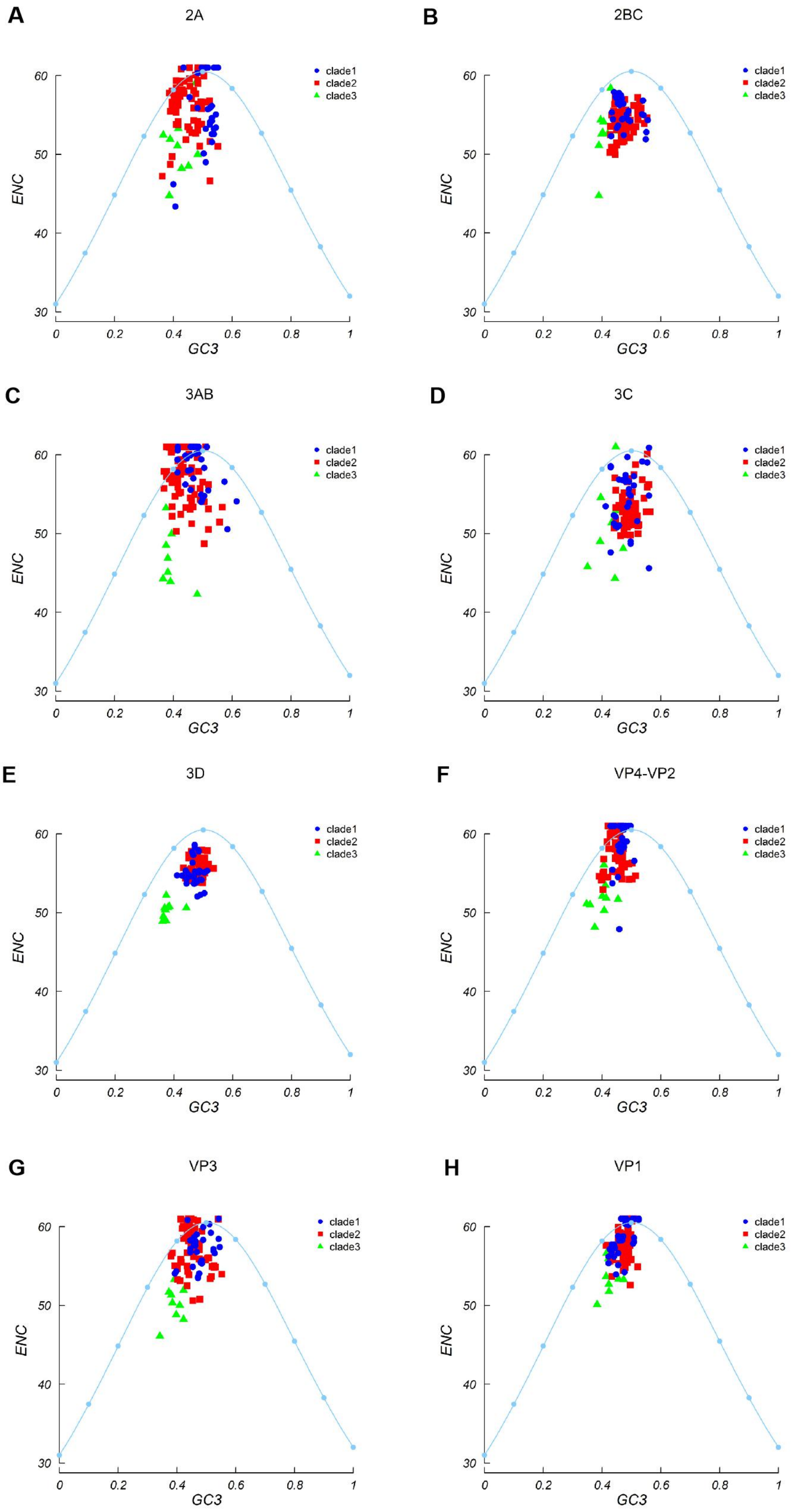


**Supplementary Figure S3.** ENC–GC3 plots for individual *EV-A* coding sequences. (A) 2A, (B) 2BC, (C) 3AB, (D) 3C, (E) 3D, (F)VP4-VP2, (G) VP3 and (H) VP1.
