# Supplementary material for: Enterovirus A Shows Unique Patterns of Codon Usage Bias in Conventional Versus Unconventional Clade
Source: Front Cell Infect Microbiol. 2022 Jul 14;12:941325. doi: 10.3389/fcimb.2022.941325 (PMC9329520; doi:10.3389/fcimb.2022.941325)
Supplement: Supplementary Figure 4 — Neutrality plot analysis. [file DataSheet_4.pdf]

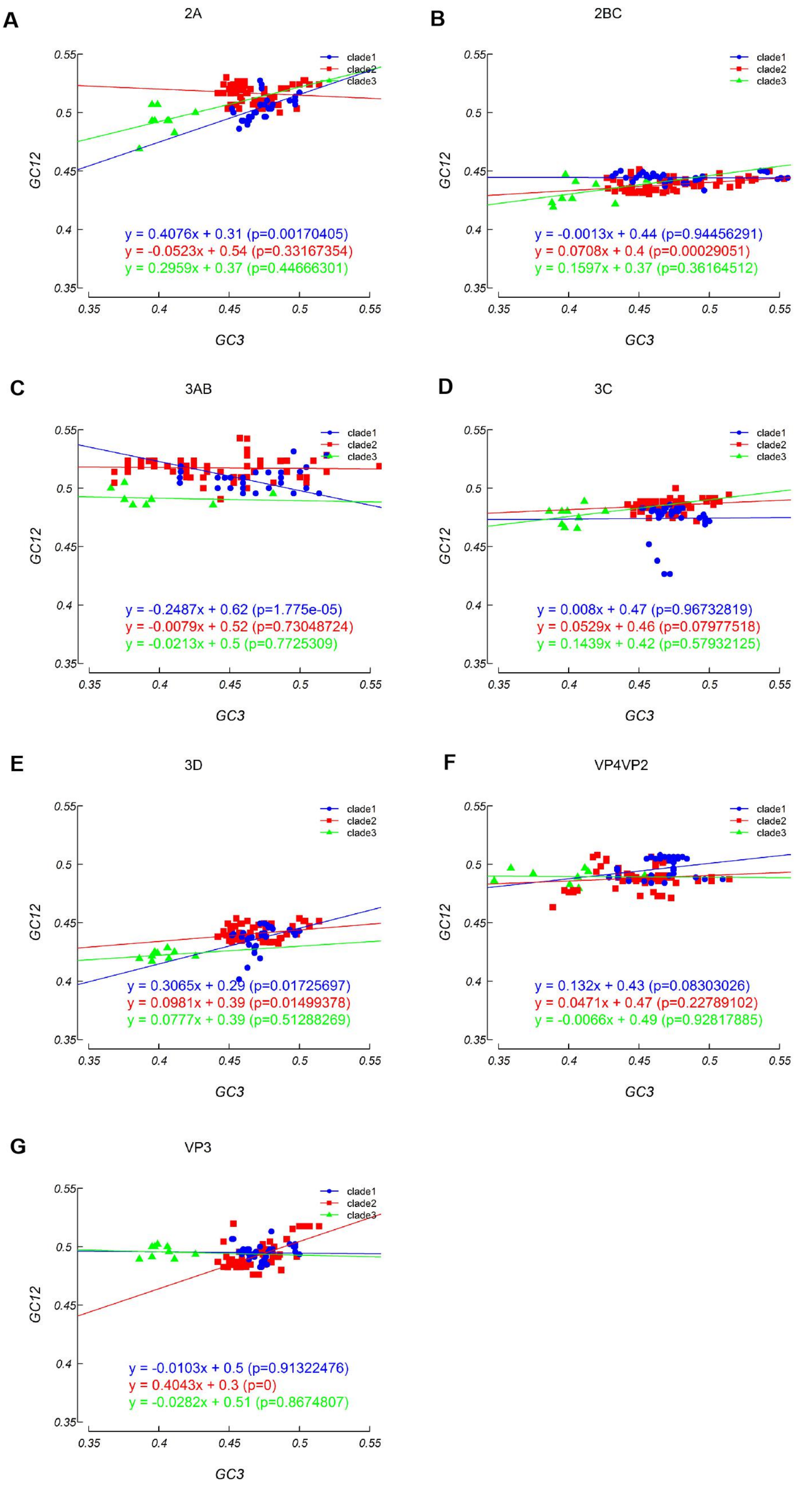

Supplementary Figure S4. Neutrality plot analysis. Neutrality plot (GC12 & GC3) was constructed for the individual genes of *EV-A* strains. (A) 2A, (B) 2BC, (C)3AB, (D) 3C, (E) 3D, (F) VP4-VP2 and (G) VP3.
