# Supplementary material for: Enterovirus A Shows Unique Patterns of Codon Usage Bias in Conventional Versus Unconventional Clade
Source: Front Cell Infect Microbiol. 2022 Jul 14;12:941325. doi: 10.3389/fcimb.2022.941325 (PMC9329520; doi:10.3389/fcimb.2022.941325)
Supplement: Supplementary Figure 5 — RCDI and SiD analysis of the EV-A coding sequences against model organisms. [file DataSheet_5.pdf]

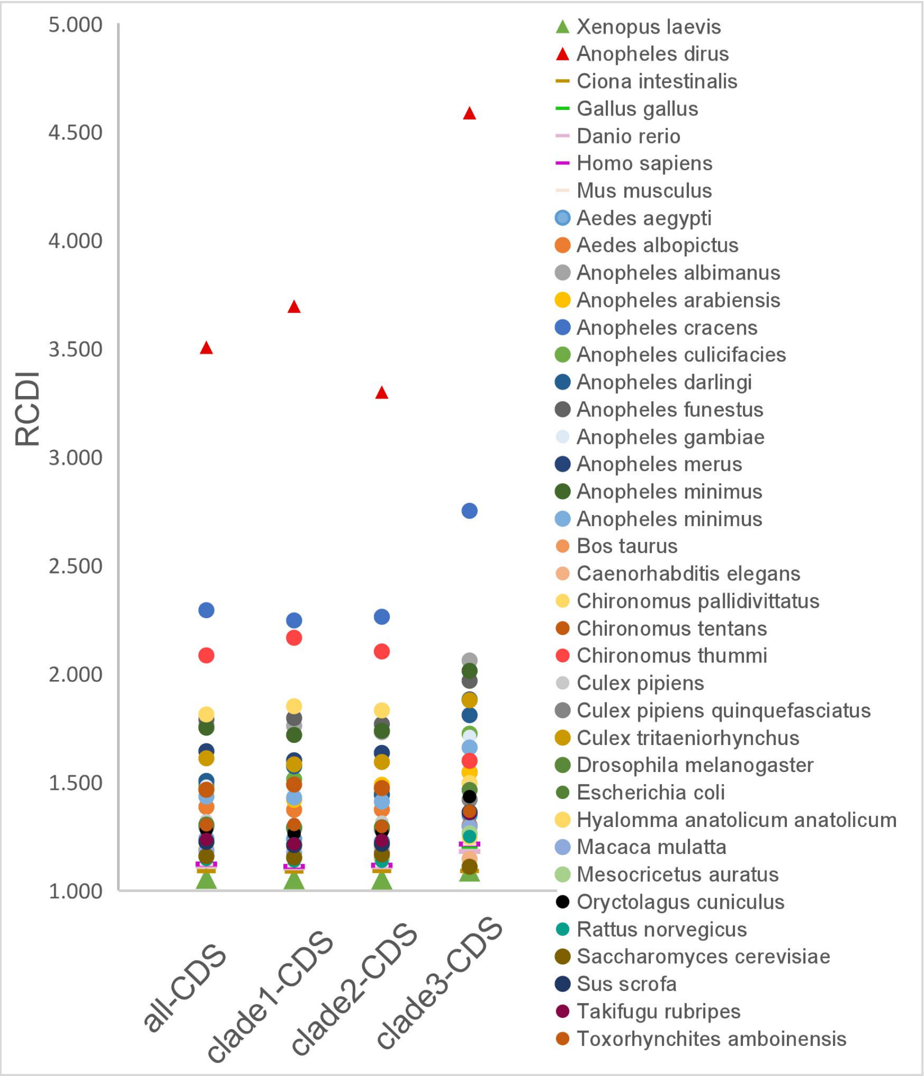

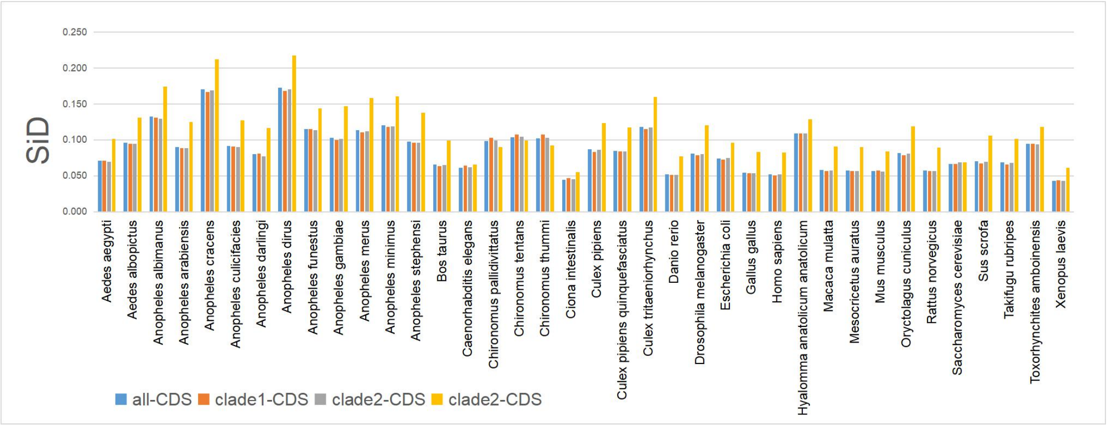

Supplementary Figure S5. RCDI and SiD analysis of the *EV-A* coding sequences against model organisms. CDS stands for the coding sequences of *EV-A* strains.
