# Supplementary material for: Enterovirus A Shows Unique Patterns of Codon Usage Bias in Conventional Versus Unconventional Clade
Source: Front Cell Infect Microbiol. 2022 Jul 14;12:941325. doi: 10.3389/fcimb.2022.941325 (PMC9329520; doi:10.3389/fcimb.2022.941325)
Supplement: Supplementary Table 1 — Demographics of Enterovirus A genomes analyzed in present study. [file Table_1.doc]

Supplementary Table S1: Demographics of *Enterovirus A* genomes analyzed in present study

| S.  NO | GenBank Accession | Collection year | GenBank Host | Virus Type | Country | Genotype^1^ |
| --- | --- | --- | --- | --- | --- | --- |
| 1 | AY421769 | 2004 | Homo sapiens | Coxsackievirus A14 | USA | clade1 |
| 2 | KP036482 | 2012 | -N/A- | Coxsackievirus A14 | China | clade1 |
| 3 | KP036483 | 2012 | -N/A- | Coxsackievirus A14 | China | clade1 |
| 4 | KC755231 | 2010 | Homo sapiens | Coxsackievirus A16 | China | clade1 |
| 5 | JX068830 | 2011 | Homo sapiens | Coxsackievirus A16 | China | clade1 |
| 6 | JQ746659 | 2015 | Homo sapiens | Coxsackievirus A16 | Malaysia | clade1 |
| 7 | KP289416 | 2013 | Homo sapiens | Coxsackievirus A16 | China | clade1 |
| 8 | KF924762 | 2010 | Homo sapiens | Coxsackievirus A16 | China | clade1 |
| 9 | EU812514 | 2008 | Homo sapiens | Coxsackievirus A16 | China | clade1 |
| 10 | U05876 | 1994 | Homo sapiens | Coxsackievirus A16 | Finland | clade1 |
| 11 | FJ198212 | 2008 | Homo sapiens | Coxsackievirus A16 | China | clade1 |
| 12 | JQ746668 | 2006 | Homo sapiens | Coxsackievirus A16 | Malaysia | clade1 |
| 13 | JQ746672 | 2007 | Homo sapiens | Coxsackievirus A16 | Malaysia | clade1 |
| 14 | JQ746676 | 2005 | Homo sapiens | Coxsackievirus A16 | Malaysia | clade1 |
| 15 | JQ746678 | 2006 | Homo sapiens | Coxsackievirus A16 | Malaysia | clade1 |
| 16 | KM516102 | 2010 | Homo sapiens | Coxsackievirus A16 | China | clade1 |
| 17 | HQ269389 | 2009 | Homo sapiens | Coxsackievirus A16 | China | clade1 |
| 18 | KY674980 | 2016 | Homo sapiens | Coxsackievirus A16 | USA | clade1 |
| 19 | KX595292 | 2014 | Homo sapiens | Coxsackievirus A16 | China | clade1 |
| 20 | KX372336 | 2012 | Homo sapiens | Coxsackievirus A16 | Thailand | clade1 |
| 21 | MF189180 | 2016 | Homo sapiens | Coxsackievirus A16 | USA | clade1 |
| 22 | GU942821 | 2012 | Homo sapiens | Coxsackievirus A7 | Finland | clade1 |
| 23 | JQ041367 | 2011 | Homo sapiens | Coxsackievirus A7 | Russia | clade1 |
| 24 | AY421765 | 2004 | Homo sapiens | Coxsackievirus A7 | USA | clade1 |
| 25 | LK021688 | 2011 | Homo sapiens | Enterovirus A120 | Madagascar | clade1 |
| 26 | MG214681 | 2015 | Homo sapiens | Enterovirus A71 | China | clade1 |
| 27 | KM077140 | 2013 | Homo sapiens | Enterovirus A71 | Taiwan | clade1 |
| 28 | AB550333 | 2001 | MOUSE L929 CELL | Enterovirus A71 | Japan | clade1 |
| 29 | DQ341357 | 2014 | Homo sapiens | Enterovirus A71 | Australia | clade1 |
| 30 | AB204852 | 2008 | Homo sapiens | Enterovirus A71 | Japan | clade1 |
| 31 | AB204853 | 2008 | Homo sapiens | Enterovirus A71 | Japan | clade1 |
| 32 | HQ647170 | 2007 | Homo sapiens | Enterovirus A71 | Canada | clade1 |
| 33 | GU434678 | 2010 | Homo sapiens | Enterovirus A71 | China | clade1 |
| 34 | KF501389 | 2010 | Homo sapiens | Enterovirus A71 | China | clade1 |
| 35 | HQ647168 | 2007 | Homo sapiens | Enterovirus A71 | Canada | clade1 |
| 36 | KF514880 | 2012 | mouse NIH/3T3 cells | Enterovirus A71 | -N/A- | clade1 |
| 37 | AY421767 | 2004 | Homo sapiens | Coxsackievirus A10 | USA | clade2 |
| 38 | KX768166 | 2015 | Homo sapiens | Coxsackievirus A10 | China | clade2 |
| 39 | KX595287 | 2013 | Homo sapiens | Coxsackievirus A10 | China | clade2 |
| 40 | KY272010 | 2014 | Homo sapiens | Coxsackievirus A10 | China | clade2 |
| 41 | KF422143 | 2011 | Homo sapiens | Coxsackievirus A12 | China | clade2 |
| 42 | KF422142 | 2009 | Homo sapiens | Coxsackievirus A12 | China | clade2 |
| 43 | AY421768 | 2004 | Homo sapiens | Coxsackievirus A12 | USA | clade2 |
| 44 | KP289360 | 2013 | Homo sapiens | Coxsackievirus A2 | China | clade2 |
| 45 | AY421760 | 2004 | Homo sapiens | Coxsackievirus A2 | USA | clade2 |
| 46 | MG214257 | 2013 | Homo sapiens | Coxsackievirus A2 | China | clade2 |
| 47 | KX595282 | 2013 | Homo sapiens | Coxsackievirus A2 | China | clade2 |
| 48 | KX595283 | 2013 | Homo sapiens | Coxsackievirus A2 | China | clade2 |
| 49 | KX595284 | 2015 | Homo sapiens | Coxsackievirus A2 | China | clade2 |
| 50 | KX595281 | 2012 | Homo sapiens | Coxsackievirus A2 | China | clade2 |
| 51 | MF678310 | 2010 | Homo sapiens | Coxsackievirus A2 | Australia | clade2 |
| 52 | MF678338 | 2008 | Homo sapiens | Coxsackievirus A2 | Australia | clade2 |
| 53 | KX810065 | 2014 | Homo sapiens | Coxsackievirus A2 | USA | clade2 |
| 54 | AY421761 | 2004 | Homo sapiens | Coxsackievirus A3 | USA | clade2 |
| 55 | KJ541164 | 2010 | Homo sapiens | Coxsackievirus A4 | China | clade2 |
| 56 | KJ541163 | 2010 | Homo sapiens | Coxsackievirus A4 | China | clade2 |
| 57 | KP676984 | 2011 | Homo sapiens | Coxsackievirus A4 | China | clade2 |
| 58 | KP676985 | 2011 | Homo sapiens | Coxsackievirus A4 | China | clade2 |
| 59 | KP676986 | 2011 | Homo sapiens | Coxsackievirus A4 | China | clade2 |
| 60 | HQ728260 | 2009 | Homo sapiens | Coxsackievirus A4 | China | clade2 |
| 61 | AY421762 | 2004 | Homo sapiens | Coxsackievirus A4 | USA | clade2 |
| 62 | KT353722 | 2008 | Homo sapiens | Coxsackievirus A4 | Taiwan | clade2 |
| 63 | KY271949 | 2015 | Homo sapiens | Coxsackievirus A4 | USA | clade2 |
| 64 | KP289362 | 2013 | Homo sapiens | Coxsackievirus A5 | China | clade2 |
| 65 | KP289363 | 2013 | Homo sapiens | Coxsackievirus A5 | China | clade2 |
| 66 | KP289364 | 2013 | Homo sapiens | Coxsackievirus A5 | China | clade2 |
| 67 | HQ728261 | 2009 | Homo sapiens | Coxsackievirus A5 | China | clade2 |
| 68 | AY421763 | 2004 | Homo sapiens | Coxsackievirus A5 | USA | clade2 |
| 69 | KU761262 | 2015 | Homo sapiens | Coxsackievirus A5 | Hungary | clade2 |
| 70 | KJ541169 | 2012 | Homo sapiens | Coxsackievirus A6 | China | clade2 |
| 71 | KJ541157 | 2012 | Homo sapiens | Coxsackievirus A6 | China | clade2 |
| 72 | KJ541158 | 2013 | Homo sapiens | Coxsackievirus A6 | China | clade2 |
| 73 | KM279379 | 2013 | Homo sapiens | Coxsackievirus A6 | China | clade2 |
| 74 | KP289367 | 2013 | Homo sapiens | Coxsackievirus A6 | China | clade2 |
| 75 | KP289369 | 2013 | Homo sapiens | Coxsackievirus A6 | China | clade2 |
| 76 | KP289370 | 2013 | Homo sapiens | Coxsackievirus A6 | China | clade2 |
| 77 | KP289371 | 2013 | Homo sapiens | Coxsackievirus A6 | China | clade2 |
| 78 | KP289372 | 2013 | Homo sapiens | Coxsackievirus A6 | China | clade2 |
| 79 | KP289376 | 2013 | Homo sapiens | Coxsackievirus A6 | China | clade2 |
| 80 | KP289384 | 2013 | Homo sapiens | Coxsackievirus A6 | China | clade2 |
| 81 | KP289385 | 2013 | Homo sapiens | Coxsackievirus A6 | China | clade2 |
| 82 | AY421764 | 2004 | Homo sapiens | Coxsackievirus A6 | USA | clade2 |
| 83 | AB779615 | 2009 | Homo sapiens | Coxsackievirus A6 | Japan | clade2 |
| 84 | AB779618 | 2009 | Homo sapiens | Coxsackievirus A6 | Japan | clade2 |
| 85 | KF682362 | 2013 | Homo sapiens | Coxsackievirus A6 | China | clade2 |
| 86 | JN582001 | 2011 | Homo sapiens | Coxsackievirus A6 | Taiwan | clade2 |
| 87 | JQ946055 | 2010 | Homo sapiens | Coxsackievirus A6 | Taiwan | clade2 |
| 88 | KT779410 | 2013 | Homo sapiens | Coxsackievirus A6 | China | clade2 |
| 89 | KX430796 | 2014 | Homo sapiens | Coxsackievirus A6 | Viet Nam | clade2 |
| 90 | KX595285 | 2014 | Homo sapiens | Coxsackievirus A6 | China | clade2 |
| 91 | KX595286 | 2014 | Homo sapiens | Coxsackievirus A6 | China | clade2 |
| 92 | KY126089 | 2013 | Homo sapiens | Coxsackievirus A6 | China | clade2 |
| 93 | KY126090 | 2013 | Homo sapiens | Coxsackievirus A6 | China | clade2 |
| 94 | KX189185 | 2011 | Homo sapiens | Coxsackievirus A6 | China | clade2 |
| 95 | KX189192 | 2013 | Homo sapiens | Coxsackievirus A6 | China | clade2 |
| 96 | KX189193 | 2013 | Homo sapiens | Coxsackievirus A6 | China | clade2 |
| 97 | KX189194 | 2013 | Homo sapiens | Coxsackievirus A6 | China | clade2 |
| 98 | LC126151 | 2009 | Homo sapiens | Coxsackievirus A6 | Japan | clade2 |
| 99 | LC126152 | 2009 | Homo sapiens | Coxsackievirus A6 | Japan | clade2 |
| 100 | LC126162 | 2013 | Homo sapiens | Coxsackievirus A6 | Japan | clade2 |
| 101 | LC126163 | 2013 | Homo sapiens | Coxsackievirus A6 | Japan | clade2 |
| 102 | KY126091 | 2014 | Homo sapiens | Coxsackievirus A6 | China | clade2 |
| 103 | MF678318 | 2012 | Homo sapiens | Coxsackievirus A6 | Australia | clade2 |
| 104 | KX064293 | 2014 | Homo sapiens | Coxsackievirus A6 | China | clade2 |
| 105 | KX064291 | 2014 | Homo sapiens | Coxsackievirus A6 | China | clade2 |
| 106 | KX064290 | 2014 | Homo sapiens | Coxsackievirus A6 | China | clade2 |
| 107 | KX064294 | 2015 | Homo sapiens | Coxsackievirus A6 | China | clade2 |
| 108 | KX064308 | 2015 | Homo sapiens | Coxsackievirus A6 | China | clade2 |
| 109 | KX064307 | 2015 | Homo sapiens | Coxsackievirus A6 | China | clade2 |
| 110 | KX064296 | 2015 | Homo sapiens | Coxsackievirus A6 | China | clade2 |
| 111 | KX064297 | 2015 | Homo sapiens | Coxsackievirus A6 | China | clade2 |
| 112 | KY126092 | 2015 | Homo sapiens | Coxsackievirus A6 | China | clade2 |
| 113 | KM609476 | 2012 | Homo sapiens | Coxsackievirus A8 | China | clade2 |
| 114 | KM609478 | 2012 | Homo sapiens | Coxsackievirus A8 | China | clade2 |
| 115 | KM609479 | 2012 | Homo sapiens | Coxsackievirus A8 | China | clade2 |
| 116 | AY421766 | 2004 | Homo sapiens | Coxsackievirus A8 | USA | clade2 |
| 117 | JF905564 | 2004 | Homo sapiens | Enterovirus A76 | China | clade3 |
| 118 | AY697458 | 2005 | Homo sapiens | Enterovirus A76 | USA | clade3 |
| 119 | AY697459 | 2005 | Homo sapiens | Enterovirus A89 | USA | clade3 |
| 120 | KT277550 | 2011 | Homo sapiens | Enterovirus A89 | China | clade3 |
| 121 | JX390655 | 2001 | Homo sapiens | Enterovirus A90 | China | clade3 |
| 122 | AY697460 | 2005 | Homo sapiens | Enterovirus A90 | USA | clade3 |
| 123 | AY773285 | 2004 | Homo sapiens | Enterovirus A90 | Netherlands | clade3 |
| 124 | AY697461 | 2005 | Homo sapiens | Enterovirus A91 | USA | clade3 |
| 125 | EF667344 | 2008 | Homo sapiens | Enterovirus A92 | USA | clade3 |

^1^; inferred from the phylogenetic analysis (Supplementary Figure S1)
