# Supplementary material for: Enterovirus A Shows Unique Patterns of Codon Usage Bias in Conventional Versus Unconventional Clade
Source: Front Cell Infect Microbiol. 2022 Jul 14;12:941325. doi: 10.3389/fcimb.2022.941325 (PMC9329520; doi:10.3389/fcimb.2022.941325)
Supplement: Supplementary Table 2 — Nucleotide composition analysis of EV-A coding sequences. [file Table_2.doc]

Supplementary Table S2: Nucleotide composition analysis of EV-A coding sequences (%)

| NO | A | U | C | G | A_3_ | | U_3_ | | C_3_ | | G_3_ | | GC | | GC_1_ | | GC_2_ | | GC_3_ | | GC_12_ | AU | | AU_3_ | |
| --- | --- | --- | --- | --- | --- | --- | --- | --- | --- | --- | --- | --- | --- | --- | --- | --- | --- | --- | --- | --- | --- | --- | --- | --- | --- |
| 1 | 28.61 | 24.78 | 24.02 | 22.59 | 25.52 | | 29.18 | | 26.71 | | 18.58 | | 46.61 | | 51.95 | | 42.59 | | 45.29 | | 47.27 | 53.39 | | 54.71 | |
| 2 | 28.61 | 24.78 | 24.02 | 22.59 | 25.52 | | 29.18 | | 26.71 | | 18.58 | | 46.61 | | 51.95 | | 42.59 | | 45.29 | | 47.27 | 53.39 | | 54.71 | |
| 3 | 28.63 | 24.78 | 24.00 | 22.56 | 25.62 | | 29.13 | | 26.66 | | 18.54 | | 46.56 | | 51.90 | | 42.59 | | 45.20 | | 47.24 | 53.41 | | 54.75 | |
| 4 | 28.33 | 24.03 | 24.98 | 22.64 | 25.06 | | 27.76 | | 28.19 | | 18.98 | | 47.62 | | 53.58 | | 42.10 | | 47.18 | | 47.84 | 52.37 | | 52.82 | |
| 5 | 28.00 | 24.23 | 25.05 | 22.71 | 24.38 | | 27.89 | | 28.89 | | 18.83 | | 47.75 | | 53.08 | | 42.46 | | 47.72 | | 47.77 | 52.23 | | 52.28 | |
| 6 | 28.07 | 24.21 | 25.06 | 22.66 | 24.57 | | 27.89 | | 28.89 | | 18.64 | | 47.72 | | 53.08 | | 42.55 | | 47.53 | | 47.82 | 52.28 | | 52.47 | |
| 7 | 27.95 | 24.29 | 24.87 | 22.90 | 23.35 | | 28.75 | | 28.75 | | 19.14 | | 47.77 | | 53.01 | | 42.40 | | 47.89 | | 47.70 | 52.23 | | 52.11 | |
| 8 | 28.44 | 24.66 | 23.84 | 23.05 | 24.63 | | 28.94 | | 26.53 | | 19.90 | | 46.90 | | 52.06 | | 42.21 | | 46.42 | | 47.13 | 53.10 | | 53.58 | |
| 9 | 28.42 | 24.67 | 23.96 | 22.95 | 24.51 | | 29.16 | | 26.70 | | 19.63 | | 46.91 | | 52.20 | | 42.20 | | 46.33 | | 47.20 | 53.09 | | 53.67 | |
| 10 | 27.88 | 24.30 | 24.87 | 22.95 | 23.22 | | 28.72 | | 28.72 | | 19.34 | | 47.82 | | 53.03 | | 42.37 | | 48.06 | | 47.70 | 52.18 | | 51.94 | |
| 11 | 28.14 | 24.82 | 23.83 | 23.21 | 23.92 | | 29.28 | | 26.62 | | 20.18 | | 47.04 | | 52.16 | | 42.16 | | 46.80 | | 47.16 | 52.96 | | 53.20 | |
| 12 | 28.18 | 24.77 | 23.87 | 23.18 | 24.03 | | 29.15 | | 26.73 | | 20.09 | | 47.05 | | 52.09 | | 42.23 | | 46.82 | | 47.16 | 52.95 | | 53.18 | |
| 13 | 28.10 | 24.83 | 23.82 | 23.24 | 23.84 | | 29.38 | | 26.54 | | 20.24 | | 47.06 | | 52.13 | | 42.27 | | 46.78 | | 47.20 | 52.94 | | 53.22 | |
| 14 | 28.09 | 24.81 | 23.83 | 23.27 | 23.83 | | 29.28 | | 26.58 | | 20.32 | | 47.10 | | 52.16 | | 42.25 | | 46.90 | | 47.21 | 52.90 | | 53.10 | |
| 15 | 28.29 | 24.80 | 23.87 | 23.04 | 24.13 | | 29.49 | | 26.46 | | 19.91 | | 46.91 | | 52.16 | | 42.20 | | 46.37 | | 47.18 | 53.09 | | 53.63 | |
| 16 | 28.50 | 24.69 | 23.93 | 22.89 | 24.61 | | 29.02 | | 26.79 | | 19.58 | | 46.82 | | 51.97 | | 42.11 | | 46.37 | | 47.04 | 53.18 | | 53.63 | |
| 17 | 28.40 | 24.81 | 23.87 | 22.92 | 24.51 | | 29.63 | | 26.32 | | 19.54 | | 46.78 | | 52.30 | | 42.20 | | 45.85 | | 47.25 | 53.22 | | 54.15 | |
| 18 | 28.40 | 24.64 | 23.94 | 23.02 | 24.38 | | 29.03 | | 26.76 | | 19.83 | | 46.96 | | 52.13 | | 42.17 | | 46.58 | | 47.15 | 53.04 | | 53.42 | |
| 19 | 28.37 | 24.68 | 23.94 | 23.02 | 24.35 | | 29.33 | | 26.58 | | 19.74 | | 46.95 | | 52.06 | | 42.48 | | 46.32 | | 47.27 | 53.05 | | 53.68 | |
| 20 | 28.06 | 24.25 | 24.85 | 22.83 | 23.45 | | 28.75 | | 28.66 | | 19.14 | | 47.69 | | 53.01 | | 42.25 | | 47.80 | | 47.63 | 52.31 | | 52.20 | |
| 21 | 28.42 | 24.64 | 23.99 | 22.95 | 24.51 | | 29.49 | | 26.36 | | 19.63 | | 46.94 | | 52.58 | | 42.25 | | 45.99 | | 47.42 | 53.06 | | 54.01 | |
| 22 | 28.49 | 24.75 | 23.85 | 22.90 | 24.72 | | 29.55 | | 26.23 | | 19.45 | | 46.74 | | 52.32 | | 42.22 | | 45.68 | | 47.27 | 53.24 | | 54.27 | |
| 23 | 28.26 | 24.32 | 24.25 | 23.17 | 23.99 | | 28.50 | | 27.12 | | 20.39 | | 47.42 | | 52.58 | | 42.15 | | 47.51 | | 47.37 | 52.58 | | 52.49 | |
| 24 | 28.09 | 24.51 | 24.01 | 23.39 | 23.66 | | 28.73 | | 26.74 | | 20.86 | | 47.40 | | 52.39 | | 42.20 | | 47.61 | | 47.30 | 52.60 | | 52.39 | |
| 25 | 28.20 | 24.41 | 24.27 | 23.13 | 24.83 | | 27.91 | | 27.44 | | 19.81 | | 47.39 | | 52.23 | | 42.70 | | 47.25 | | 47.46 | 52.61 | | 52.75 | |
| 26 | 28.20 | 24.39 | 24.28 | 23.13 | 24.79 | | 27.87 | | 27.49 | | 19.86 | | 47.41 | | 52.23 | | 42.65 | | 47.35 | | 47.44 | 52.59 | | 52.65 | |
| 27 | 27.84 | 23.76 | 24.71 | 23.70 | 23.98 | | 26.35 | | 28.63 | | 21.04 | | 48.40 | | 52.75 | | 42.80 | | 49.67 | | 47.77 | 51.60 | | 50.33 | |
| 28 | 27.81 | 23.85 | 24.64 | 23.71 | 23.77 | | 26.52 | | 28.50 | | 21.21 | | 48.34 | | 52.75 | | 42.57 | | 49.72 | | 47.66 | 51.66 | | 50.28 | |
| 29 | 28.19 | 24.44 | 24.25 | 23.12 | 24.77 | | 27.95 | | 27.48 | | 19.80 | | 47.37 | | 52.20 | | 42.63 | | 47.28 | | 47.42 | 52.63 | | 52.72 | |
| 30 | 28.24 | 24.37 | 24.27 | 23.12 | 24.86 | | 27.84 | | 27.46 | | 19.84 | | 47.40 | | 52.08 | | 42.80 | | 47.30 | | 47.44 | 52.60 | | 52.70 | |
| 31 | 27.44 | 24.44 | 24.62 | 23.50 | 23.26 | | 28.71 | | 27.71 | | 20.32 | | 48.11 | | 53.43 | | 42.87 | | 48.03 | | 48.15 | 51.89 | | 51.97 | |
| 32 | 27.74 | 23.98 | 24.52 | 23.76 | 23.74 | | 26.97 | | 28.15 | | 21.14 | | 48.28 | | 52.75 | | 42.80 | | 49.29 | | 47.77 | 51.72 | | 50.71 | |
| 33 | 27.50 | 23.93 | 24.66 | 23.90 | 22.99 | | 26.97 | | 28.39 | | 21.66 | | 48.56 | | 52.65 | | 42.99 | | 50.05 | | 47.82 | 51.44 | | 49.95 | |
| 34 | 27.71 | 23.97 | 24.61 | 23.71 | 23.65 | | 26.78 | | 28.53 | | 21.04 | | 48.33 | | 52.61 | | 42.80 | | 49.57 | | 47.70 | 51.67 | | 50.43 | |
| 35 | 27.69 | 23.97 | 24.60 | 23.74 | 23.60 | | 26.68 | | 28.58 | | 21.14 | | 48.34 | | 52.61 | | 42.70 | | 49.72 | | 47.65 | 51.66 | | 50.28 | |
| 36 | 28.40 | 24.15 | 24.53 | 22.92 | 25.64 | | 27.21 | | 27.49 | | 19.66 | | 47.45 | | 52.94 | | 42.26 | | 47.15 | | 47.60 | 52.55 | | 52.85 | |
| 37 | 27.94 | 23.68 | 25.30 | 23.08 | 23.62 | | 26.93 | | 29.63 | | 19.83 | | 48.38 | | 53.57 | | 42.12 | | 49.46 | | 47.85 | 51.62 | | 50.54 | |
| 38 | 27.71 | 23.44 | 25.53 | 23.33 | 23.31 | | 26.34 | | 30.02 | | 20.33 | | 48.86 | | 53.95 | | 42.27 | | 50.35 | | 48.11 | 51.14 | | 49.65 | |
| 39 | 27.69 | 23.48 | 25.44 | 23.39 | 23.12 | | 26.57 | | 29.79 | | 20.52 | | 48.83 | | 54.00 | | 42.17 | | 50.31 | | 48.09 | 51.17 | | 49.69 | |
| 40 | 27.69 | 23.46 | 25.56 | 23.30 | 22.91 | | 26.41 | | 30.24 | | 20.44 | | 48.86 | | 53.72 | | 42.17 | | 50.69 | | 47.94 | 51.14 | | 49.31 | |
| 41 | 27.47 | 23.37 | 25.59 | 23.57 | 22.42 | | 26.16 | | 30.27 | | 21.14 | | 49.16 | | 53.83 | | 42.24 | | 51.42 | | 48.04 | 50.84 | | 48.58 | |
| 42 | 28.08 | 24.76 | 24.12 | 23.04 | 24.38 | | 30.30 | | 25.76 | | 19.55 | | 47.16 | | 53.93 | | 42.23 | | 45.31 | | 48.08 | 52.84 | | 54.69 | |
| 43 | 28.06 | 24.34 | 24.38 | 23.17 | 23.77 | | 28.31 | | 27.46 | | 20.36 | | 47.55 | | 52.70 | | 42.14 | | 47.82 | | 47.42 | 52.40 | | 52.08 | |
| 44 | 27.74 | 23.57 | 25.37 | 23.32 | 23.18 | | 26.87 | | 29.61 | | 20.34 | | 48.69 | | 53.97 | | 42.15 | | 49.95 | | 48.06 | 51.31 | | 50.05 | |
| 45 | 27.86 | 23.91 | 24.46 | 23.71 | 23.58 | | 27.27 | | 27.56 | | 21.59 | | 48.17 | | 52.84 | | 42.52 | | 49.15 | | 47.68 | 51.77 | | 50.85 | |
| 46 | 28.49 | 24.86 | 24.15 | 22.44 | 25.71 | | 29.73 | | 26.56 | | 17.99 | | 46.59 | | 52.94 | | 42.28 | | 44.55 | | 47.61 | 53.35 | | 55.45 | |
| 47 | 28.39 | 24.60 | 24.25 | 22.75 | 24.31 | | 28.29 | | 27.49 | | 19.91 | | 47.00 | | 51.37 | | 42.23 | | 47.39 | | 46.80 | 52.99 | | 52.61 | |
| 48 | 28.57 | 24.32 | 24.41 | 22.70 | 24.27 | | 29.45 | | 27.00 | | 19.27 | | 47.11 | | 53.35 | | 41.71 | | 46.28 | | 47.53 | 52.89 | | 53.72 | |
| 49 | 28.63 | 24.61 | 24.07 | 22.69 | 24.67 | | 29.47 | | 26.74 | | 19.11 | | 46.77 | | 52.73 | | 41.71 | | 45.86 | | 47.22 | 53.23 | | 54.14 | |
| 50 | 28.77 | 24.78 | 23.89 | 22.57 | 25.09 | | 29.99 | | 26.27 | | 18.64 | | 46.45 | | 52.73 | | 41.71 | | 44.92 | | 47.22 | 53.55 | | 55.08 | |
| 51 | 28.56 | 24.71 | 23.90 | 22.83 | 24.49 | | 29.96 | | 26.10 | | 19.45 | | 46.73 | | 52.99 | | 41.64 | | 45.55 | | 47.32 | 53.27 | | 54.45 | |
| 52 | 28.59 | 24.67 | 23.89 | 22.85 | 24.52 | | 30.13 | | 26.12 | | 19.24 | | 46.74 | | 53.18 | | 41.68 | | 45.36 | | 47.43 | 53.26 | | 54.64 | |
| 53 | 28.54 | 24.65 | 23.96 | 22.83 | 24.54 | | 30.05 | | 26.19 | | 19.17 | | 46.79 | | 53.27 | | 41.73 | | 45.36 | | 47.50 | 53.20 | | 54.59 | |
| 54 | 28.67 | 24.59 | 24.15 | 22.59 | 24.82 | | 29.39 | | 26.90 | | 18.89 | | 46.74 | | 52.76 | | 41.69 | | 45.78 | | 47.22 | 53.26 | | 54.22 | |
| 55 | 28.78 | 24.67 | 23.82 | 22.72 | 24.91 | | 29.43 | | 26.27 | | 19.40 | | 46.55 | | 52.21 | | 41.76 | | 45.67 | | 46.99 | 53.45 | | 54.33 | |
| 56 | 28.66 | 24.76 | 23.94 | 22.62 | 24.63 | | 30.25 | | 26.19 | | 18.88 | | 46.56 | | 52.95 | | 41.67 | | 45.07 | | 47.31 | 53.42 | | 54.88 | |
| 57 | 27.84 | 24.57 | 24.00 | 23.60 | 23.46 | | 29.05 | | 26.26 | | 21.23 | | 47.60 | | 52.42 | | 42.89 | | 47.49 | | 47.65 | 52.40 | | 52.51 | |
| 58 | 28.18 | 24.02 | 24.34 | 23.47 | 24.24 | | 27.23 | | 27.85 | | 20.68 | | 47.80 | | 52.23 | | 42.65 | | 48.53 | | 47.44 | 52.20 | | 51.47 | |
| 59 | 28.33 | 23.84 | 24.51 | 23.32 | 24.58 | | 26.86 | | 28.19 | | 20.36 | | 47.82 | | 52.16 | | 42.76 | | 48.55 | | 47.46 | 52.18 | | 51.45 | |
| 60 | 28.27 | 24.13 | 24.27 | 23.32 | 24.24 | | 27.70 | | 27.66 | | 20.40 | | 47.60 | | 51.99 | | 42.74 | | 48.06 | | 47.37 | 52.40 | | 51.94 | |
| 61 | 28.26 | 24.12 | 24.23 | 23.39 | 24.42 | | 27.50 | | 27.60 | | 20.48 | | 47.62 | | 52.06 | | 42.72 | | 48.08 | | 47.39 | 52.38 | | 51.92 | |
| 62 | 28.32 | 24.30 | 24.02 | 23.37 | 24.68 | | 27.72 | | 27.10 | | 20.50 | | 47.38 | | 51.92 | | 42.62 | | 47.60 | | 47.27 | 52.62 | | 52.40 | |
| 63 | 28.29 | 24.03 | 24.88 | 22.79 | 24.22 | | 27.62 | | 28.79 | | 19.37 | | 47.68 | | 52.31 | | 42.55 | | 48.16 | | 47.43 | 52.32 | | 51.84 | |
| 64 | 28.58 | 24.70 | 24.00 | 22.72 | 24.81 | | 29.99 | | 26.22 | | 18.97 | | 46.72 | | 52.54 | | 42.42 | | 45.20 | | 47.48 | 53.28 | | 54.80 | |
| 65 | 28.62 | 24.63 | 24.13 | 22.61 | 24.96 | | 29.82 | | 26.57 | | 18.65 | | 46.74 | | 52.43 | | 42.58 | | 45.22 | | 47.50 | 53.26 | | 54.78 | |
| 66 | 28.48 | 24.71 | 24.01 | 22.80 | 24.63 | | 30.24 | | 26.05 | | 19.08 | | 46.80 | | 52.76 | | 42.53 | | 45.12 | | 47.64 | 53.20 | | 54.88 | |
| 67 | 28.39 | 24.57 | 24.20 | 22.85 | 24.40 | | 29.63 | | 26.80 | | 19.17 | | 47.04 | | 52.57 | | 42.58 | | 45.97 | | 47.57 | 52.96 | | 54.03 | |
| 68 | 28.40 | 24.62 | 24.10 | 22.88 | 24.31 | | 29.82 | | 26.47 | | 19.41 | | 46.98 | | 52.61 | | 42.44 | | 45.88 | | 47.53 | 53.02 | | 54.12 | |
| 69 | 28.64 | 24.78 | 23.96 | 22.63 | 25.25 | | 30.19 | | 26.10 | | 18.46 | | 46.59 | | 52.61 | | 42.58 | | 44.56 | | 47.60 | 53.41 | | 55.44 | |
| 70 | 27.93 | 24.13 | 24.81 | 23.13 | 23.79 | | 28.31 | | 28.31 | | 19.59 | | 47.94 | | 52.90 | | 43.01 | | 47.90 | | 47.95 | 52.06 | | 52.10 | |
| 71 | 28.37 | 24.56 | 24.20 | 22.88 | 24.35 | | 29.72 | | 26.71 | | 19.22 | | 47.07 | | 52.71 | | 42.58 | | 45.93 | | 47.64 | 52.93 | | 54.07 | |
| 72 | 28.47 | 24.59 | 24.13 | 22.81 | 24.59 | | 29.82 | | 26.42 | | 19.17 | | 46.95 | | 52.61 | | 42.63 | | 45.60 | | 47.62 | 53.05 | | 54.40 | |
| 73 | 28.54 | 24.49 | 24.24 | 22.72 | 24.63 | | 29.39 | | 26.94 | | 19.03 | | 46.96 | | 52.47 | | 42.44 | | 45.97 | | 47.46 | 53.04 | | 54.03 | |
| 74 | 28.40 | 24.68 | 24.09 | 22.83 | 24.40 | | 30.05 | | 26.33 | | 19.22 | | 46.91 | | 52.66 | | 42.53 | | 45.55 | | 47.60 | 53.09 | | 54.45 | |
| 75 | 28.54 | 24.51 | 24.26 | 22.69 | 24.68 | | 29.58 | | 26.85 | | 18.89 | | 46.95 | | 52.61 | | 42.49 | | 45.74 | | 47.55 | 53.05 | | 54.26 | |
| 76 | 28.50 | 24.80 | 24.02 | 22.68 | 24.93 | | 30.25 | | 26.20 | | 18.61 | | 46.70 | | 52.54 | | 42.74 | | 44.82 | | 47.64 | 53.30 | | 55.18 | |
| 77 | 28.56 | 24.51 | 24.21 | 22.72 | 24.82 | | 29.63 | | 26.66 | | 18.89 | | 46.93 | | 52.66 | | 42.58 | | 45.55 | | 47.62 | 53.07 | | 54.45 | |
| 78 | 28.37 | 24.60 | 24.16 | 22.86 | 24.31 | | 29.82 | | 26.61 | | 19.27 | | 47.02 | | 52.57 | | 42.63 | | 45.88 | | 47.60 | 52.98 | | 54.12 | |
| 79 | 27.97 | 24.20 | 24.72 | 23.12 | 23.87 | | 28.67 | | 27.92 | | 19.54 | | 47.83 | | 53.06 | | 42.98 | | 47.46 | | 48.02 | 52.17 | | 52.54 | |
| 80 | 28.43 | 24.56 | 24.15 | 22.86 | 24.35 | | 29.67 | | 26.61 | | 19.36 | | 47.01 | | 52.47 | | 42.58 | | 45.97 | | 47.53 | 52.99 | | 54.03 | |
| 81 | 28.51 | 24.51 | 24.21 | 22.77 | 24.63 | | 29.53 | | 26.75 | | 19.08 | | 46.98 | | 52.57 | | 42.53 | | 45.83 | | 47.55 | 53.02 | | 54.17 | |
| 82 | 28.48 | 24.63 | 24.10 | 22.78 | 24.63 | | 29.91 | | 26.38 | | 19.08 | | 46.88 | | 52.61 | | 42.58 | | 45.45 | | 47.60 | 53.12 | | 54.55 | |
| 83 | 28.62 | 24.92 | 23.82 | 22.64 | 25.01 | | 30.76 | | 25.58 | | 18.65 | | 46.46 | | 52.57 | | 42.58 | | 44.23 | | 47.57 | 53.54 | | 55.77 | |
| 84 | 28.54 | 24.35 | 24.37 | 22.74 | 24.73 | | 29.16 | | 27.13 | | 18.98 | | 47.10 | | 52.61 | | 42.58 | | 46.11 | | 47.60 | 52.90 | | 53.89 | |
| 85 | 28.45 | 24.56 | 24.15 | 22.83 | 24.44 | | 29.76 | | 26.55 | | 19.26 | | 46.99 | | 52.64 | | 42.51 | | 45.81 | | 47.58 | 53.01 | | 54.19 | |
| 86 | 28.26 | 24.65 | 24.16 | 22.92 | 24.12 | | 29.96 | | 26.52 | | 19.41 | | 47.09 | | 52.80 | | 42.53 | | 45.93 | | 47.67 | 52.91 | | 54.07 | |
| 87 | 28.26 | 24.47 | 24.33 | 22.94 | 24.06 | | 29.47 | | 27.02 | | 19.44 | | 47.27 | | 52.82 | | 42.51 | | 46.47 | | 47.67 | 52.73 | | 53.53 | |
| 88 | 28.59 | 24.59 | 24.18 | 22.64 | 24.87 | | 29.72 | | 26.71 | | 18.70 | | 46.82 | | 52.52 | | 42.53 | | 45.41 | | 47.53 | 53.18 | | 54.59 | |
| 89 | 28.47 | 24.56 | 24.20 | 22.77 | 24.58 | | 29.66 | | 26.69 | | 19.07 | | 46.97 | | 52.59 | | 42.56 | | 45.76 | | 47.58 | 53.03 | | 54.24 | |
| 90 | 28.47 | 24.59 | 24.15 | 22.80 | 24.54 | | 29.67 | | 26.66 | | 19.12 | | 46.95 | | 52.43 | | 42.63 | | 45.78 | | 47.53 | 53.05 | | 54.22 | |
| 91 | 28.40 | 24.62 | 24.15 | 22.83 | 24.45 | | 29.96 | | 26.47 | | 19.12 | | 46.98 | | 52.71 | | 42.63 | | 45.60 | | 47.67 | 53.02 | | 54.40 | |
| 92 | 28.56 | 24.46 | 24.25 | 22.71 | 24.79 | | 29.31 | | 26.96 | | 18.90 | | 46.97 | | 52.50 | | 42.55 | | 45.85 | | 47.53 | 53.02 | | 54.10 | |
| 93 | 28.59 | 24.67 | 24.09 | 22.66 | 24.96 | | 29.91 | | 26.47 | | 18.65 | | 46.74 | | 52.52 | | 42.58 | | 45.12 | | 47.55 | 53.26 | | 54.88 | |
| 94 | 28.39 | 24.73 | 24.05 | 22.84 | 24.36 | | 30.16 | | 26.30 | | 19.18 | | 46.89 | | 52.59 | | 42.60 | | 45.48 | | 47.60 | 53.11 | | 54.52 | |
| 95 | 28.43 | 24.63 | 24.13 | 22.80 | 24.49 | | 29.91 | | 26.42 | | 19.17 | | 46.93 | | 52.57 | | 42.63 | | 45.60 | | 47.60 | 53.07 | | 54.40 | |
| 96 | 28.43 | 24.67 | 24.10 | 22.80 | 24.40 | | 29.96 | | 26.42 | | 19.22 | | 46.90 | | 52.52 | | 42.53 | | 45.64 | | 47.53 | 53.10 | | 54.36 | |
| 97 | 28.37 | 24.56 | 24.16 | 22.91 | 24.31 | | 29.58 | | 26.71 | | 19.41 | | 47.07 | | 52.47 | | 42.63 | | 46.11 | | 47.55 | 52.93 | | 53.89 | |
| 98 | 28.48 | 24.43 | 24.29 | 22.80 | 24.59 | | 29.25 | | 27.08 | | 19.08 | | 47.09 | | 52.57 | | 42.53 | | 46.16 | | 47.55 | 52.91 | | 53.84 | |
| 99 | 28.46 | 24.66 | 24.20 | 22.68 | 24.66 | | 29.89 | | 26.69 | | 18.76 | | 46.88 | | 52.66 | | 42.53 | | 45.45 | | 47.60 | 53.12 | | 54.55 | |
| 100 | 28.52 | 24.67 | 24.16 | 22.65 | 24.75 | | 29.89 | | 26.59 | | 18.76 | | 46.80 | | 52.52 | | 42.53 | | 45.36 | | 47.52 | 53.20 | | 54.64 | |
| 101 | 28.40 | 24.73 | 23.96 | 22.92 | 24.51 | | 30.30 | | 25.87 | | 19.32 | | 46.87 | | 52.78 | | 42.65 | | 45.19 | | 47.71 | 53.13 | | 54.81 | |
| 102 | 28.35 | 24.76 | 23.96 | 22.93 | 24.46 | | 30.40 | | 25.87 | | 19.27 | | 46.89 | | 52.92 | | 42.60 | | 45.15 | | 47.76 | 53.11 | | 54.85 | |
| 103 | 28.52 | 24.67 | 24.06 | 22.76 | 24.91 | | 29.99 | | 26.27 | | 18.83 | | 46.81 | | 52.78 | | 42.56 | | 45.10 | | 47.67 | 53.19 | | 54.90 | |
| 104 | 28.50 | 24.87 | 23.91 | 22.73 | 24.60 | | 30.40 | | 26.01 | | 18.99 | | 46.64 | | 52.36 | | 42.55 | | 45.00 | | 47.46 | 53.36 | | 55.00 | |
| 105 | 28.48 | 24.87 | 23.91 | 22.75 | 24.65 | | 30.40 | | 26.01 | | 18.94 | | 46.65 | | 52.45 | | 42.55 | | 44.96 | | 47.50 | 53.35 | | 55.04 | |
| 106 | 28.28 | 23.52 | 24.94 | 23.26 | 23.90 | | 26.32 | | 29.03 | | 20.76 | | 48.20 | | 52.68 | | 42.14 | | 49.79 | | 47.41 | 51.80 | | 50.21 | |
| 107 | 28.70 | 24.24 | 24.40 | 22.66 | 24.86 | | 28.32 | | 27.75 | | 19.07 | | 47.06 | | 52.56 | | 41.79 | | 46.82 | | 47.18 | 52.94 | | 53.18 | |
| 108 | 28.68 | 24.34 | 24.40 | 22.56 | 24.57 | | 28.32 | | 27.94 | | 19.12 | | 46.96 | | 52.13 | | 41.70 | | 47.06 | | 46.92 | 53.02 | | 52.89 | |
| 109 | 28.73 | 24.29 | 24.37 | 22.61 | 24.86 | | 28.46 | | 27.66 | | 19.02 | | 46.98 | | 52.51 | | 41.75 | | 46.68 | | 47.13 | 53.02 | | 53.32 | |
| 110 | 28.17 | 24.29 | 24.32 | 23.22 | 23.91 | | 28.46 | | 27.51 | | 20.12 | | 47.54 | | 52.46 | | 42.52 | | 47.63 | | 47.49 | 52.46 | | 52.37 | |
| 111 | 28.14 | 24.49 | 24.01 | 23.36 | 23.96 | | 28.88 | | 26.70 | | 20.45 | | 47.36 | | 52.41 | | 42.52 | | 47.16 | | 47.47 | 52.64 | | 52.84 | |
| 112 | 28.33 | 24.37 | 24.08 | 23.22 | 24.48 | | 28.13 | | 27.08 | | 20.31 | | 47.30 | | 51.85 | | 42.66 | | 47.40 | | 47.25 | 52.70 | | 52.60 | |
| 113 | 28.17 | 24.45 | 24.10 | 23.25 | 24.01 | | 28.88 | | 26.94 | | 20.17 | | 47.35 | | 52.18 | | 42.76 | | 47.11 | | 47.47 | 52.62 | | 52.89 | |
| 114 | 28.28 | 24.05 | 24.30 | 23.37 | 23.95 | | 28.47 | | 27.14 | | 20.44 | | 47.67 | | 53.18 | | 42.25 | | 47.58 | | 47.72 | 52.33 | | 52.42 | |
| 115 | 28.52 | 23.74 | 24.53 | 23.21 | 24.32 | | 26.94 | | 28.46 | | 20.29 | | 47.74 | | 52.02 | | 42.47 | | 48.74 | | 47.24 | 52.26 | | 51.26 | |
| 116 | 28.23 | 23.92 | 24.45 | 23.38 | 23.86 | | 28.18 | | 27.57 | | 20.39 | | 47.83 | | 53.37 | | 42.16 | | 47.96 | | 47.77 | 52.15 | | 52.04 | |
| 117 | 30.23 | 25.15 | 23.40 | 21.13 | 28.04 | | 30.73 | | 25.30 | | 15.74 | | 44.54 | | 50.97 | | 41.61 | | 41.04 | | 46.29 | 55.38 | | 58.77 | |
| 118 | 30.94 | 25.55 | 23.04 | 20.47 | 30.07 | | 31.35 | | 24.68 | | 13.90 | | 43.51 | | 50.69 | | 41.28 | | 38.58 | | 45.98 | 56.49 | | 61.42 | |
| 119 | 30.21 | 25.77 | 22.94 | 21.08 | 28.56 | | 31.98 | | 24.25 | | 15.21 | | 44.02 | | 51.30 | | 41.31 | | 39.46 | | 46.31 | 55.98 | | 60.54 | |
| 120 | 30.35 | 25.49 | 23.13 | 21.03 | 28.85 | | 31.26 | | 24.63 | | 15.25 | | 44.17 | | 51.59 | | 41.02 | | 39.89 | | 46.31 | 55.83 | | 60.11 | |
| 121 | 29.71 | 25.45 | 23.82 | 20.99 | 27.36 | | 31.91 | | 25.56 | | 15.13 | | 44.81 | | 51.87 | | 41.87 | | 40.68 | | 46.87 | 55.16 | | 59.27 | |
| 122 | 29.54 | 24.94 | 24.32 | 21.19 | 26.84 | | 30.54 | | 27.03 | | 15.60 | | 45.52 | | 52.20 | | 41.73 | | 42.63 | | 46.97 | 54.48 | | 57.37 | |
| 123 | 30.05 | 25.40 | 23.81 | 20.74 | 28.48 | | 31.90 | | 25.59 | | 14.03 | | 44.55 | | 52.13 | | 41.90 | | 39.62 | | 47.01 | 55.45 | | 60.38 | |
| 124 | 30.38 | 25.46 | 23.41 | 20.74 | 29.12 | | 31.34 | | 25.24 | | 14.25 | | 44.14 | | 51.14 | | 41.81 | | 39.49 | | 46.47 | 55.84 | | 60.46 | |
| 125 | 30.64 | 24.96 | 23.00 | 21.40 | 29.25 | | 30.10 | | 25.74 | | 14.91 | | 44.40 | | 50.43 | | 42.12 | | 40.65 | | 46.27 | 55.60 | | 59.35 | |
| Summary table | | | | | |  | |  | |  | |  | |  | |  | |  | |  | | |  | |  |
|  | A | U | C | G | A_3_ | | U_3_ | | C_3_ | | G_3_ | | GC | | GC_1_ | | GC_2_ | | GC_3_ | | GC_12_ | AU | | AU_3_ | |
| clade1 | 28±0.3 | 24±0.3 | 24±0.4 | 23±0.4 | 24±0.7 | | 28±1.0 | | 27±0.9 | | 20±0.8 | | 47±0.6 | | 52±0.4 | | 42±0.3 | | 47±1.3 | | 47±0.3 | 53±0.6 | | 53±1.3 | |
| clade2 | 28±0.3 | 24±0.4 | 24±0.4 | 23±0.3 | 24±0.5 | | 29±1.2 | | 27±1.1 | | 19±0.7 | | 47±0.6 | | 53±0.5 | | 42±0.3 | | 47±1.6 | | 48±0.2 | 53±0.6 | | 53±1.6 | |
| clade3 | 30±0.4 | 25±0.3 | 23±0.5 | 21±0.3 | 29±1.0 | | 31±0.7 | | 25±0.8 | | 15±0.7 | | 44±0.6 | | 51±0.6 | | 42±0.4 | | 40±1.2 | | 46±0.4 | 56±0.6 | | 60±1.2 | |
| All clade | 28±0.6 | 24±0.4 | 24±0.5 | 23±0.6 | 25±1.2 | | 29±1.3 | | 27±1.1 | | 19±1.4 | | 47±1.0 | | 53±0.6 | | 42±0.4 | | 46±2.3 | | 47±0.4 | 53±1.0 | | 54±2.3 | |

The numbering of EV-A strains in first column corresponds to that of Supplementary Table S1. Summary table shows the average value(Avg) and standard deviation(SD) of Nucleotide composition in specfic clade and all clade.
