# Supplementary material for: Enterovirus A Shows Unique Patterns of Codon Usage Bias in Conventional Versus Unconventional Clade
Source: Front Cell Infect Microbiol. 2022 Jul 14;12:941325. doi: 10.3389/fcimb.2022.941325 (PMC9329520; doi:10.3389/fcimb.2022.941325)
Supplement: Supplementary Table 3 — One way Anova analysis between three clades. [file Table_3.doc]

Supplementary Table S3: One way Anova analysis between three clades

|  | D-value of mean value(%) | | | F values between clades | Significance between caldes |
| --- | --- | --- | --- | --- | --- |
|  | clade1-clade2 | clade1-clade3 | clade2 -clade3 |  |  |
| A | -0.20857* | -2.06972** | -1.86115** | 183.041 | 0.000 |
| U | 0.01876 | -0.91583** | -0.9346** | 27.973 | 0.000 |
| G | 0.00649 | 0.86111** | 0.85463** | 18.227 | 0.000 |
| C | 0.18493* | 2.13861** | 1.95368** | 176.659 | 0.000 |
| A3 | -0.09632 | -4.22472** | -4.12840** | 186.06 | 0.000 |
| U3 | -0.65729* | -2.81861** | -2.16132** | 23.183 | 0.000 |
| G3 | 0.35292 | 2.08361** | 1.73069** | 15.258 | 0.000 |
| C3 | 0.40060* | 4.98611** | 4.58551** | 169.824 | 0.000 |
| GC | 0.19131 | 2.99639** | 2.80508** | 98.296 | 0.000 |
| GC1 | -0.21585* | 1.10639** | 1.32224** | 30.333 | 0.000 |
| GC2 | 0.03831 | 0.81028** | 0.77197** | 25.213 | 0.000 |
| GC3 | 0.75239* | 7.06972** | 6.31733** | 82.002 | 0.000 |
| GC12 | -0.08999 | 0.95861** | 1.04860** | 64.525 | 0.000 |
| AU | -0.18990 | -2.98417** | -2.79426** | 97.408 | 0.000 |
| AU3 | -0.75142* | -7.04028** | -6.28886** | 81.321 | 0.000 |

1. Value is short for the difference between two numbers

F-Value is a test value of Anova analysis.The analysis is finished by SPSS 23.0.

^*^:P-value≤0.05

^**^:P-value≤0.01
