# Supplementary material for: Enterovirus A Shows Unique Patterns of Codon Usage Bias in Conventional Versus Unconventional Clade
Source: Front Cell Infect Microbiol. 2022 Jul 14;12:941325. doi: 10.3389/fcimb.2022.941325 (PMC9329520; doi:10.3389/fcimb.2022.941325)
Supplement: Supplementary Table 4 — Summary data of CAI, RCDI and SiD analysis. [file Table_4.doc]

**Supplementary Table S4.** Summary data of CAI, RCDI and SiD analysis.

**Table S4A. Summary of codon adaptation index (CAI) analysis.**

|  | **All**  **CDS** | **All**  **P1** | **All**  **p2p3** | **Clade1**  **CDS** | **Clade1**  **P1** | **Clade1**  **P2P3** | **Clade2**  **CDS** | **Clade2**  **P1** | **Clade2_p2p3** | **Clade3CDS** | **Clade3P1** | **Clade3P2P3** |
| --- | --- | --- | --- | --- | --- | --- | --- | --- | --- | --- | --- | --- |
| *Aedes aegypti* | 0.705 | 0.698 | 0.710 | 0.703 | 0.695 | 0.709 | 0.706 | 0.699 | 0.711 | 0.702 | 0.704 | 0.705 |
| *Aedes albopictus* | 0.621 | 0.610 | 0.628 | 0.623 | 0.611 | 0.630 | 0.621 | 0.610 | 0.629 | 0.608 | 0.600 | 0.614 |
| *Anopheles albimanus* | 0.500 | 0.489 | 0.506 | 0.502 | 0.493 | 0.508 | 0.500 | 0.489 | 0.508 | 0.480 | 0.475 | 0.483 |
| *Anopheles arabiensis* | 0.627 | 0.615 | 0.635 | 0.632 | 0.619 | 0.641 | 0.626 | 0.615 | 0.633 | 0.623 | 0.608 | 0.633 |
| *Anopheles cracens* | 0.413 | 0.391 | 0.428 | 0.420 | 0.399 | 0.434 | 0.413 | 0.391 | 0.429 | 0.388 | 0.366 | 0.402 |
| *Anopheles culicifacies* | 0.621 | 0.608 | 0.630 | 0.619 | 0.607 | 0.626 | 0.624 | 0.609 | 0.633 | 0.613 | 0.551 | 0.618 |
| *Anopheles darlingi* | 0.653 | 0.645 | 0.659 | 0.648 | 0.640 | 0.652 | 0.657 | 0.648 | 0.664 | 0.641 | 0.640 | 0.576 |
| *Anopheles dirus* | 0.355 | 0.334 | 0.370 | 0.358 | 0.339 | 0.372 | 0.357 | 0.334 | 0.373 | 0.327 | 0.307 | 0.341 |
| *Anopheles funestus* | 0.554 | 0.534 | 0.568 | 0.551 | 0.533 | 0.562 | 0.556 | 0.535 | 0.570 | 0.550 | 0.523 | 0.568 |
| *Anopheles gambiae* | 0.571 | 0.559 | 0.579 | 0.576 | 0.565 | 0.583 | 0.571 | 0.558 | 0.580 | 0.552 | 0.539 | 0.560 |
| *Anopheles merus* | 0.525 | 0.503 | 0.540 | 0.530 | 0.509 | 0.544 | 0.525 | 0.503 | 0.540 | 0.507 | 0.479 | 0.525 |
| *Anopheles minimus* | 0.561 | 0.539 | 0.576 | 0.564 | 0.545 | 0.576 | 0.561 | 0.538 | 0.577 | 0.547 | 0.523 | 0.563 |
| *Anopheles stephensi* | 0.611 | 0.599 | 0.619 | 0.613 | 0.602 | 0.620 | 0.612 | 0.599 | 0.621 | 0.594 | 0.582 | 0.602 |
| *Bos taurus* | 0.679 | 0.670 | 0.685 | 0.681 | 0.670 | 0.688 | 0.679 | 0.670 | 0.684 | 0.670 | 0.669 | 0.671 |
| *Caenorhabditis elegans* | 0.674 | 0.662 | 0.683 | 0.666 | 0.651 | 0.676 | 0.674 | 0.662 | 0.682 | 0.709 | 0.699 | 0.715 |
| *Chironomus pallidivittatus* | 0.507 | 0.483 | 0.524 | 0.497 | 0.474 | 0.513 | 0.505 | 0.479 | 0.523 | 0.569 | 0.548 | 0.584 |
| *Chironomus tentans* | 0.555 | 0.530 | 0.573 | 0.546 | 0.522 | 0.563 | 0.553 | 0.527 | 0.571 | 0.608 | 0.583 | 0.624 |
| *Chironomus thummi* | 0.479 | 0.447 | 0.502 | 0.467 | 0.437 | 0.487 | 0.477 | 0.443 | 0.501 | 0.546 | 0.517 | 0.566 |
| *Ciona intestinalis* | 0.778 | 0.772 | 0.782 | 0.772 | 0.766 | 0.777 | 0.777 | 0.772 | 0.781 | 0.803 | 0.796 | 0.808 |
| *Culex pipiens* | 0.635 | 0.618 | 0.645 | 0.638 | 0.621 | 0.650 | 0.635 | 0.619 | 0.645 | 0.620 | 0.602 | 0.631 |
| *Culex pipiens*  *quinquefasciatus* | 0.645 | 0.633 | 0.653 | 0.645 | 0.631 | 0.654 | 0.646 | 0.635 | 0.654 | 0.635 | 0.624 | 0.642 |
| *Culex tritaeniorhynchus* | 0.539 | 0.522 | 0.550 | 0.544 | 0.528 | 0.554 | 0.539 | 0.522 | 0.550 | 0.517 | 0.499 | 0.530 |
| *Danio rerio* | 0.764 | 0.761 | 0.766 | 0.763 | 0.758 | 0.767 | 0.764 | 0.761 | 0.766 | 0.766 | 0.773 | 0.761 |
| *Drosophila melanogaster* | 0.637 | 0.633 | 0.641 | 0.640 | 0.635 | 0.643 | 0.638 | 0.632 | 0.641 | 0.626 | 0.626 | 0.626 |
| *Escherichia coli* | 0.730 | 0.739 | 0.725 | 0.733 | 0.740 | 0.728 | 0.728 | 0.737 | 0.722 | 0.742 | 0.750 | 0.738 |
| *Gallus gallus* | 0.772 | 0.771 | 0.773 | 0.773 | 0.769 | 0.775 | 0.772 | 0.772 | 0.773 | 0.770 | 0.776 | 0.766 |
| *Homo sapiens* | 0.742 | 0.733 | 0.748 | 0.743 | 0.731 | 0.750 | 0.742 | 0.734 | 0.747 | 0.740 | 0.739 | 0.741 |
| *Hyalomma anatolicum anatolicum* | 0.728 | 0.718 | 0.734 | 0.722 | 0.712 | 0.728 | 0.729 | 0.719 | 0.736 | 0.739 | 0.733 | 0.744 |
| *Macaca mulatta* | 0.679 | 0.672 | 0.683 | 0.680 | 0.669 | 0.687 | 0.679 | 0.673 | 0.684 | 0.670 | 0.673 | 0.668 |
| *Mesocricetus auratus* | 0.696 | 0.688 | 0.702 | 0.697 | 0.685 | 0.704 | 0.697 | 0.689 | 0.703 | 0.690 | 0.691 | 0.688 |
| *Mus musculus* | 0.740 | 0.733 | 0.744 | 0.740 | 0.730 | 0.746 | 0.740 | 0.734 | 0.744 | 0.737 | 0.740 | 0.734 |
| *Oryctolagus cuniculus* | 0.600 | 0.592 | 0.606 | 0.604 | 0.593 | 0.611 | 0.601 | 0.592 | 0.606 | 0.586 | 0.583 | 0.587 |
| *Rattus norvegicus* | 0.711 | 0.705 | 0.716 | 0.712 | 0.703 | 0.718 | 0.712 | 0.706 | 0.716 | 0.705 | 0.708 | 0.703 |
| *Saccharomyces cerevisiae* | 0.711 | 0.703 | 0.716 | 0.705 | 0.697 | 0.710 | 0.709 | 0.702 | 0.715 | 0.745 | 0.739 | 0.749 |
| *Sus scrofa* | 0.639 | 0.630 | 0.646 | 0.642 | 0.630 | 0.650 | 0.639 | 0.630 | 0.646 | 0.627 | 0.624 | 0.628 |
| *Takifugu rubripes* | 0.649 | 0.644 | 0.652 | 0.653 | 0.646 | 0.657 | 0.648 | 0.644 | 0.652 | 0.636 | 0.640 | 0.634 |
| *Toxorhynchites*  *amboinensis* | 0.663 | 0.644 | 0.675 | 0.661 | 0.644 | 0.672 | 0.662 | 0.642 | 0.676 | 0.674 | 0.662 | 0.683 |
| *Xenopus laevis* | 0.815 | 0.803 | 0.823 | 0.810 | 0.794 | 0.821 | 0.816 | 0.805 | 0.823 | 0.829 | 0.823 | 0.832 |

**Table S4B. Summary of relative codon deoptimization index (RCDI) analysis.**

|  | **All**  **CDS** | **All**  **P1** | **All**  **p2p3** | **Clade1**  **CDS** | **Clade1**  **P1** | **Clade1**  **P2P3** | **Clade2**  **CDS** | **Clade2**  **P1** | **Clade2_p2p3** | **Clade3CDS** | **Clade3P1** | **Clade3P2P3** |
| --- | --- | --- | --- | --- | --- | --- | --- | --- | --- | --- | --- | --- |
| *Aedes aegypti* | 1.239 | 1.277 | 1.261 | 1.237 | 1.267 | 1.264 | 1.227 | 1.273 | 1.246 | 1.348 | 1.350 | 1.384 |
| *Aedes albopictus* | 1.386 | 1.428 | 1.410 | 1.373 | 1.409 | 1.400 | 1.374 | 1.422 | 1.394 | 1.546 | 1.547 | 1.588 |
| *Anopheles albimanus* | 1.763 | 1.812 | 1.800 | 1.760 | 1.781 | 1.812 | 1.731 | 1.803 | 1.755 | 2.062 | 2.010 | 2.149 |
| *Anopheles arabiensis* | 1.472 | 1.552 | 1.470 | 1.418 | 1.515 | 1.406 | 1.488 | 1.560 | 1.493 | 1.545 | 1.632 | 1.532 |
| *Anopheles cracens* | 2.294 | 2.421 | 2.293 | 2.246 | 2.360 | 2.256 | 2.263 | 2.406 | 2.254 | 2.752 | 2.802 | 2.789 |
| *Anopheles culicifacies* | 1.487 | 1.510 | 1.533 | 1.514 | 1.533 | 1.561 | 1.448 | 1.479 | 1.491 | 1.724 | 1.685 | 1.797 |
| *Anopheles darlingi* | 1.506 | 1.517 | 1.560 | 1.574 | 1.575 | 1.633 | 1.442 | 1.466 | 1.489 | 1.810 | 1.745 | 1.904 |
| *Anopheles dirus* | 3.507 | 3.650 | 3.844 | 3.696 | 3.844 | 3.746 | 3.300 | 3.457 | 3.337 | 4.589 | 4.581 | 4.715 |
| *Anopheles funestus* | 1.791 | 1.822 | 1.832 | 1.796 | 1.808 | 1.847 | 1.768 | 1.803 | 1.810 | 1.968 | 2.051 | 1.963 |
| *Anopheles gambiae* | 1.482 | 1.513 | 1.514 | 1.462 | 1.483 | 1.501 | 1.465 | 1.504 | 1.494 | 1.711 | 1.716 | 1.754 |
| *Anopheles merus* | 1.643 | 1.712 | 1.655 | 1.601 | 1.673 | 1.611 | 1.635 | 1.702 | 1.649 | 1.882 | 1.962 | 1.879 |
| *Anopheles minimus* | 1.752 | 1.754 | 1.811 | 1.718 | 1.717 | 1.780 | 1.737 | 1.743 | 1.795 | 2.014 | 1.990 | 2.080 |
| *Anopheles stephensi* | 1.434 | 1.478 | 1.458 | 1.429 | 1.462 | 1.459 | 1.411 | 1.463 | 1.431 | 1.660 | 1.672 | 1.697 |
| *Bos taurus* | 1.179 | 1.228 | 1.192 | 1.165 | 1.219 | 1.176 | 1.171 | 1.222 | 1.184 | 1.299 | 1.312 | 1.327 |
| *Caenorhabditis elegans* | 1.193 | 1.242 | 1.207 | 1.201 | 1.245 | 1.218 | 1.195 | 1.248 | 1.208 | 1.146 | 1.178 | 1.160 |
| *Chironomus pallidivittatus* | 1.813 | 1.926 | 1.822 | 1.851 | 1.906 | 1.890 | 1.831 | 1.980 | 1.825 | 1.496 | 1.530 | 1.526 |
| *Chironomus tentans* | 1.465 | 1.546 | 1.474 | 1.489 | 1.547 | 1.511 | 1.473 | 1.569 | 1.476 | 1.300 | 1.342 | 1.314 |
| *Chironomus thummi* | 2.085 | 2.341 | 2.020 | 2.166 | 2.307 | 2.165 | 2.103 | 2.431 | 2.001 | 1.599 | 1.674 | 1.606 |
| *Ciona intestinalis* | 1.090 | 1.107 | 1.118 | 1.089 | 1.105 | 1.117 | 1.091 | 1.108 | 1.120 | 1.090 | 1.110 | 1.110 |
| *Culex pipiens* | 1.323 | 1.387 | 1.332 | 1.299 | 1.366 | 1.307 | 1.316 | 1.382 | 1.326 | 1.472 | 1.516 | 1.486 |
| *Culex pipiens*  *quinquefasciatus* | 1.294 | 1.351 | 1.307 | 1.286 | 1.343 | 1.300 | 1.283 | 1.344 | 1.295 | 1.420 | 1.454 | 1.440 |
| *Culex tritaeniorhynchus* | 1.611 | 1.674 | 1.626 | 1.582 | 1.637 | 1.604 | 1.593 | 1.664 | 1.604 | 1.878 | 1.916 | 1.902 |
| *Danio rerio* | 1.113 | 1.160 | 1.126 | 1.105 | 1.151 | 1.118 | 1.110 | 1.160 | 1.121 | 1.181 | 1.192 | 1.208 |
| *Drosophila melanogaster* | 1.305 | 1.341 | 1.329 | 1.292 | 1.319 | 1.322 | 1.292 | 1.337 | 1.312 | 1.464 | 1.455 | 1.508 |
| *Escherichia coli* | 1.221 | 1.226 | 1.263 | 1.204 | 1.205 | 1.246 | 1.221 | 1.228 | 1.264 | 1.283 | 1.284 | 1.318 |
| *Gallus gallus* | 1.113 | 1.152 | 1.129 | 1.103 | 1.146 | 1.118 | 1.107 | 1.147 | 1.123 | 1.201 | 1.213 | 1.228 |
| *Homo sapiens* | 1.123 | 1.168 | 1.136 | 1.112 | 1.163 | 1.122 | 1.117 | 1.164 | 1.131 | 1.215 | 1.229 | 1.240 |
| *Hyalomma anatolicum anatolicum* | 1.192 | 1.240 | 1.206 | 1.212 | 1.258 | 1.229 | 1.176 | 1.229 | 1.188 | 1.247 | 1.265 | 1.271 |
| *Macaca mulatta* | 1.183 | 1.234 | 1.196 | 1.174 | 1.235 | 1.181 | 1.175 | 1.225 | 1.189 | 1.295 | 1.305 | 1.327 |
| *Mesocricetus auratus* | 1.157 | 1.211 | 1.167 | 1.150 | 1.211 | 1.157 | 1.148 | 1.204 | 1.157 | 1.265 | 1.275 | 1.294 |
| *Mus musculus* | 1.121 | 1.169 | 1.132 | 1.113 | 1.167 | 1.123 | 1.113 | 1.164 | 1.125 | 1.214 | 1.225 | 1.241 |
| *Oryctolagus cuniculus* | 1.281 | 1.337 | 1.294 | 1.264 | 1.326 | 1.274 | 1.272 | 1.329 | 1.285 | 1.433 | 1.450 | 1.464 |
| *Rattus norvegicus* | 1.144 | 1.192 | 1.156 | 1.136 | 1.190 | 1.147 | 1.135 | 1.186 | 1.147 | 1.250 | 1.259 | 1.279 |
| *Saccharomyces cerevisiae* | 1.159 | 1.182 | 1.193 | 1.153 | 1.171 | 1.186 | 1.167 | 1.192 | 1.202 | 1.110 | 1.134 | 1.129 |
| *Sus scrofa* | 1.225 | 1.278 | 1.237 | 1.210 | 1.269 | 1.220 | 1.216 | 1.271 | 1.228 | 1.361 | 1.376 | 1.391 |
| *Takifugu rubripes* | 1.236 | 1.287 | 1.249 | 1.215 | 1.267 | 1.229 | 1.231 | 1.287 | 1.242 | 1.358 | 1.368 | 1.391 |
| *Toxorhynchites*  *amboinensis* | 1.303 | 1.366 | 1.312 | 1.305 | 1.358 | 1.320 | 1.295 | 1.367 | 1.299 | 1.366 | 1.386 | 1.391 |
| *Xenopus laevis* | 1.057 | 1.097 | 1.072 | 1.056 | 1.101 | 1.068 | 1.055 | 1.095 | 1.070 | 1.091 | 1.109 | 1.111 |

**Table S4C. Summary of Similarity index (SiD) analysis**

|  | **All**  **CDS** | **All**  **P1** | **All**  **p2p3** | **Clade1**  **CDS** | **Clade1**  **P1** | **Clade1**  **P2P3** | **Clade2**  **CDS** | **Clade2**  **P1** | **Clade2_p2p3** | **Clade3CDS** | **Clade3P1** | **Clade3P2P3** |
| --- | --- | --- | --- | --- | --- | --- | --- | --- | --- | --- | --- | --- |
| *Aedes aegypti* | 0.071 | 0.067 | 0.076 | 0.071 | 0.068 | 0.076 | 0.070 | 0.066 | 0.075 | 0.101 | 0.094 | 0.107 |
| *Aedes albopictus* | 0.096 | 0.091 | 0.101 | 0.095 | 0.092 | 0.099 | 0.095 | 0.090 | 0.100 | 0.131 | 0.122 | 0.137 |
| *Anopheles albimanus* | 0.132 | 0.126 | 0.138 | 0.131 | 0.127 | 0.136 | 0.130 | 0.125 | 0.135 | 0.174 | 0.163 | 0.181 |
| *Anopheles arabiensis* | 0.090 | 0.085 | 0.096 | 0.089 | 0.086 | 0.093 | 0.089 | 0.084 | 0.095 | 0.125 | 0.117 | 0.130 |
| *Anopheles cracens* | 0.170 | 0.163 | 0.177 | 0.166 | 0.160 | 0.173 | 0.169 | 0.163 | 0.176 | 0.212 | 0.202 | 0.219 |
| *Anopheles culicifacies* | 0.092 | 0.086 | 0.097 | 0.091 | 0.087 | 0.096 | 0.090 | 0.085 | 0.096 | 0.127 | 0.118 | 0.133 |
| *Anopheles darlingi* | 0.080 | 0.075 | 0.085 | 0.081 | 0.077 | 0.086 | 0.077 | 0.074 | 0.083 | 0.116 | 0.106 | 0.124 |
| *Anopheles dirus* | 0.172 | 0.165 | 0.179 | 0.168 | 0.163 | 0.174 | 0.171 | 0.164 | 0.177 | 0.218 | 0.207 | 0.225 |
| *Anopheles funestus* | 0.115 | 0.105 | 0.123 | 0.115 | 0.107 | 0.123 | 0.113 | 0.104 | 0.122 | 0.144 | 0.135 | 0.151 |
| *Anopheles gambiae* | 0.103 | 0.095 | 0.111 | 0.100 | 0.093 | 0.107 | 0.102 | 0.094 | 0.109 | 0.147 | 0.136 | 0.154 |
| *Anopheles merus* | 0.114 | 0.105 | 0.121 | 0.111 | 0.103 | 0.118 | 0.112 | 0.103 | 0.120 | 0.158 | 0.148 | 0.165 |
| *Anopheles minimus* | 0.120 | 0.110 | 0.129 | 0.118 | 0.110 | 0.126 | 0.119 | 0.108 | 0.128 | 0.160 | 0.149 | 0.168 |
| *Anopheles stephensi* | 0.098 | 0.091 | 0.104 | 0.096 | 0.091 | 0.102 | 0.096 | 0.090 | 0.102 | 0.137 | 0.128 | 0.144 |
| *Bos taurus* | 0.065 | 0.063 | 0.069 | 0.063 | 0.063 | 0.066 | 0.065 | 0.064 | 0.069 | 0.099 | 0.091 | 0.105 |
| *Caenorhabditis elegans* | 0.061 | 0.075 | 0.064 | 0.064 | 0.075 | 0.066 | 0.062 | 0.075 | 0.065 | 0.066 | 0.110 | 0.067 |
| *Chironomus pallidivittatus* | 0.098 | 0.099 | 0.100 | 0.103 | 0.104 | 0.104 | 0.099 | 0.101 | 0.102 | 0.090 | 0.089 | 0.091 |
| *Chironomus tentans* | 0.103 | 0.103 | 0.106 | 0.107 | 0.107 | 0.109 | 0.104 | 0.105 | 0.107 | 0.099 | 0.098 | 0.100 |
| *Chironomus thummi* | 0.102 | 0.106 | 0.103 | 0.108 | 0.111 | 0.108 | 0.103 | 0.108 | 0.104 | 0.092 | 0.092 | 0.093 |
| *Ciona intestinalis* | 0.045 | 0.042 | 0.049 | 0.046 | 0.044 | 0.051 | 0.045 | 0.044 | 0.050 | 0.055 | 0.052 | 0.058 |
| *Culex pipiens* | 0.087 | 0.079 | 0.094 | 0.083 | 0.078 | 0.089 | 0.086 | 0.078 | 0.094 | 0.124 | 0.115 | 0.130 |
| *Culex pipiens*  *quinquefasciatus* | 0.085 | 0.080 | 0.090 | 0.084 | 0.081 | 0.088 | 0.084 | 0.079 | 0.089 | 0.117 | 0.110 | 0.123 |
| *Culex tritaeniorhynchus* | 0.118 | 0.109 | 0.126 | 0.115 | 0.107 | 0.122 | 0.117 | 0.109 | 0.125 | 0.159 | 0.150 | 0.166 |
| *Danio rerio* | 0.052 | 0.051 | 0.055 | 0.051 | 0.051 | 0.054 | 0.052 | 0.052 | 0.055 | 0.077 | 0.070 | 0.083 |
| *Drosophila melanogaster* | 0.081 | 0.075 | 0.087 | 0.078 | 0.075 | 0.083 | 0.080 | 0.075 | 0.086 | 0.120 | 0.110 | 0.128 |
| *Escherichia coli* | 0.074 | 0.069 | 0.080 | 0.073 | 0.067 | 0.078 | 0.075 | 0.070 | 0.081 | 0.096 | 0.090 | 0.102 |
| *Gallus gallus* | 0.054 | 0.052 | 0.058 | 0.053 | 0.052 | 0.057 | 0.054 | 0.052 | 0.058 | 0.083 | 0.077 | 0.089 |
| *Homo sapiens* | 0.052 | 0.050 | 0.056 | 0.050 | 0.050 | 0.053 | 0.052 | 0.050 | 0.056 | 0.082 | 0.075 | 0.088 |
| *Hyalomma anatolicum anatolicum* | 0.109 | 0.107 | 0.114 | 0.109 | 0.106 | 0.113 | 0.109 | 0.108 | 0.113 | 0.129 | 0.124 | 0.133 |
| *Macaca mulatta* | 0.058 | 0.057 | 0.061 | 0.056 | 0.058 | 0.059 | 0.057 | 0.058 | 0.061 | 0.091 | 0.083 | 0.097 |
| *Mesocricetus auratus* | 0.058 | 0.056 | 0.061 | 0.056 | 0.057 | 0.059 | 0.057 | 0.057 | 0.061 | 0.090 | 0.082 | 0.096 |
| *Mus musculus* | 0.057 | 0.056 | 0.060 | 0.057 | 0.056 | 0.061 | 0.056 | 0.056 | 0.059 | 0.084 | 0.078 | 0.089 |
| *Oryctolagus cuniculus* | 0.082 | 0.080 | 0.086 | 0.079 | 0.079 | 0.082 | 0.081 | 0.080 | 0.085 | 0.119 | 0.110 | 0.126 |
| *Rattus norvegicus* | 0.058 | 0.056 | 0.061 | 0.056 | 0.057 | 0.059 | 0.057 | 0.056 | 0.061 | 0.089 | 0.082 | 0.095 |
| *Saccharomyces cerevisiae* | 0.066 | 0.065 | 0.070 | 0.066 | 0.065 | 0.070 | 0.069 | 0.068 | 0.072 | 0.068 | 0.066 | 0.072 |
| *Sus scrofa* | 0.070 | 0.068 | 0.074 | 0.067 | 0.067 | 0.070 | 0.069 | 0.068 | 0.073 | 0.106 | 0.097 | 0.112 |
| *Takifugu rubripes* | 0.069 | 0.067 | 0.072 | 0.066 | 0.065 | 0.069 | 0.068 | 0.068 | 0.072 | 0.102 | 0.093 | 0.108 |
| *Toxorhynchites*  *amboinensis* | 0.094 | 0.092 | 0.098 | 0.094 | 0.093 | 0.098 | 0.094 | 0.092 | 0.098 | 0.118 | 0.112 | 0.123 |
| *Xenopus laevis* | 0.043 | 0.042 | 0.046 | 0.044 | 0.044 | 0.047 | 0.043 | 0.043 | 0.046 | 0.061 | 0.057 | 0.065 |
